# Expression Profiling of Ribosomal Protein Gene Family in Dehydration Stress Responses and Characterization of Transgenic Rice Plants Overexpressing *RPL23A* for Water-Use Efficiency and Tolerance to Drought and Salt Stresses

**DOI:** 10.3389/fchem.2017.00097

**Published:** 2017-11-14

**Authors:** Mazahar Moin, Achala Bakshi, M. S. Madhav, P. B. Kirti

**Affiliations:** ^1^Department of Plant Sciences, University of Hyderabad, Hyderabad, India; ^2^Department of Biotechnology, Indian Institute of Rice Research, Hyderabad, India

**Keywords:** rice, *RPL23A*, ribosomal protein genes, water-use efficiency, drought stress, overexpression

## Abstract

Our previous findings on the screening of a large-pool of activation tagged rice plants grown under limited water conditions revealed the activation of Ribosomal Protein Large (RPL) subunit genes, *RPL6* and *RPL23A* in two mutants that exhibited high water-use efficiency (WUE) with the genes getting activated by the integrated 4x enhancers (Moin et al., 2016a). In continuation of these findings, we have comprehensively characterized the Ribosomal Protein (RP) gene family including both small (RPS) and large (RPL) subunits, which have been identified to be encoded by at least 70 representative genes; RP-genes exist as multiple expressed copies with high nucleotide and amino acid sequence similarity. The differential expression of all the representative genes in rice was performed under limited water and drought conditions at progressive time intervals in the present study. More than 50% of the RP genes were upregulated in both shoot and root tissues. Some of them exhibited an overlap in upregulation under both the treatments indicating that they might have a common role in inducing tolerance under limited water and drought conditions. Among the genes that became significantly upregulated in both the tissues and under both the treatments are *RPL6, 7, 23A, 24*, and *31* and *RPS4, 10* and *18a*. To further validate the role of RP genes in WUE and inducing tolerance to other stresses, we have raised transgenic plants overexpressing *RPL23A* in rice. The high expression lines of *RPL23A* exhibited low Δ^13^C, increased quantum efficiency along with suitable growth and yield parameters with respect to negative control under the conditions of limited water availability. The constitutive expression of *RPL23A* was also associated with transcriptional upregulation of many other RPL and RPS genes. The seedlings of *RPL23A* high expression lines also showed a significant increase in fresh weight, root length, proline and chlorophyll contents under simulated drought and salt stresses. Taken together, our findings provide a secure basis for the RPL gene family expression as a potential resource for exploring abiotic stress tolerant properties in rice.

## Introduction

Rice is one of the widely used monocot model crops for functional genomic studies and a primary staple cereal for more than half of the world population. It is very vulnerable to changing environmental conditions, such as water scarcity, drought, salinity and pathogen attack, which cause yield losses of more than 50% per annum (Wang et al., 2003). Of about 56,000 genes that exist in the rice genome, the functional characterization of <10% of them (~600 genes) has been undertaken for having roles in inducing tolerance to various stresses, while the functions of other genes remain to be elucidated. The more direct approach of investigating the functions of plant genes is through mutagenesis approaches (such as gain-of-function or loss-of-function) followed by independent overexpression or silencing in the transgenic plants (Moin et al., 2017). Transgenic technology has opened the vistas for the development of new varieties with improved performance under the conditions of limited resource availability.

Abiotic stress factors, such as water scarcity, drought, salinity, and pathogen attack induce the activation of a large number of genes, which are regulated by complex transcriptional networks (Yamaguchi-Shinozaki and Shinozaki, 2006). Some of the genes involved in these transcriptional networks (ABA-dependant and ABA-independent) form important candidates for the development of stress-tolerant transgenic rice. Overexpression of the transcription factors like bHLH, bZIP, NAC, AP2/ERF, MYB, Zinc finger, WRKY, and kinases in transgenic rice has resulted in increased yield under abiotic stress conditions (Dubouzet et al., 2003; Zhang et al., 2004; Karaba et al., 2007; Nakashima et al., 2007; Hu et al., 2008; Jeong et al., 2010).

Plant tolerance to water stress occurs either by drought avoidance or drought tolerance mechanisms (Blum, 2005, 2009). Drought avoidance, which is different from water-use efficiency (WUE), is the maintenance of high water status (water conservation) under water deficit conditions, and thereby, promotes WUE (Karaba et al., 2007). In physiological terms, WUE refers to the ratio of unit of water lost through transpiration in relation to the photosynthesis in the plant. In other words, WUE can be equated with grain yield and water used by crop (Blum, 2005). Drought tolerance refers to the ability of one genotype to yield better than the other in a severely dehydrated state (Blum, 2005).

Ribosomal proteins (RP) are ubiquitous in nature and are well-known for their universal roles in forming and stabilizing the ribosomal complex and mediating protein synthesis. The ribosomal complex is encoded by around 60–80 ribosomal genes in all the eukaryotes (Ban et al., 2000; Barakat et al., 2001; Hanson et al., 2004). RP-genes exist as multiple, expressed copies with high nucleotide and amino acid sequence similarity. An RP synthesized from only one gene copy of a group incorporates into a ribosome complex under a given condition or in a tissue (Guarinos et al., 2003; Schuwirth et al., 2005). This supports the fact that ribosomes are heterogeneous in nature and their peptide (expressed genic) composition tends to change in response to developmental stages, tissues and external stimuli, such as stress factors (Schmid et al., 2005; Byrne, 2009). Although RP genes exist as paralogs, all of them are differentially required for normal development, with some of them functioning in spatio-temporal manner with stimulus-induced expression, while others exhibit binding properties (Wool, 1996; Warner and McIntosh, 2009).

RP genes have been shown to be differentially regulated by environmental factors, both abiotic and biotic, which directly affect the plant growth and transcriptional regulation of RP genes and ultimately ribosome biogenesis (Fromont-Racine et al., 2003). *RPL10* was found to be significantly upregulated by UV-B radiation (Casati and Walbot, 2003; Ferreyra et al., 2010). *RPL10* has also been identified as a substrate of NIK (NSP-interacting Kinase) and functions as a downstream effector of NIK1 in plant defense against viruses (Carvalho et al., 2008). *RPL10* was also one of the genes that became significantly upregulated in treatments with *Xanthomonas oryzae* (Moin et al., 2016b) and Zhu et al. (2017) have reported the identification of the insect resistance properties of NlRPL5 in rice.

Arabidopsis *RPL23A* is a part of universally conserved r-protein located in the cytoplasm that binds directly to large subunit (LSU) rRNA and is essential for ribosome biogenesis (Lecompte et al., 2002). In yeast, RPL23aA protein binds to a specific site on the 26S rRNA and RPL23aA functionality was confirmed by its ability to complement yeast *l25* mutant (McIntosh and Bonham-Smith, 2001). This protein is also one of the target molecules involved in growth-mediated inhibition by interferons (Jiang et al., 1997). The two isoforms of Arabidopsis *RPL23A, RPL23Aa* and *RPL23Ab* have been identified with *cis*-regulatory elements in their promoter regions and are involved in transcriptional, post-transcriptional and translational regulation (McIntosh et al., 2011). A knockout of Arabidopsis *AtRPL23Aa* resulted in retarded plant growth, irregular leaf and root morphology and loss of apical dominance, and proper functioning of *RPL23A* is essential for plant viability (Degenhardt and Bonham-Smith, 2008).

The other RPL genes, such as *RPL35* and *RPL32* became up- and down-regulated in heat and salt treatments, respectively (Mukhopadhyay et al., 2011). The Arabidopsis plastid RP L11, *PRPL11* gene was upregulated by salt stress and its mutants showed pale leaves and defective growth (Omidbakhshfard et al., 2012). Mutation in *AtRPL24* negatively affected the development and also reinitiation of translation of transcription factor, bZIP11 (ATB2) and ARF mORF (Zhou et al., 2010). Similar to RPL, the RPS genes are also differentially regulated by stress (Liu and Baird, 2003; Saha et al., 2017) and mutations in some of these genes and overexpression of others caused perturbed plant phenotypes (Lijsebettens et al., 1994; Ito et al., 2000) and tolerance to stresses (Liang et al., 2015), respectively.

Taking a cue from our previous findings (Moin et al., 2016a,b), the current study has been initiated on elucidating the dehydration stress-responsive properties of ribosomal genes, both RPL and RPS. In the present study, we have presented an overview of the differential expression pattern of the representative RP genes in response to limited water and drought stress treatments under greenhouse conditions at progressive time intervals. We have investigated their overall expression patterns in shoot and root tissues at four different time points. Also, we have identified specific RP genes, whose expression is unique or overlapping under limited water and drought conditions. We have also validated the role of *RPL23A* in WUE by its overexpression in independent transgenic rice plants.

In summary, the information presented in this study provides a resource for subsequent exploitation of RP genes to ameliorate abiotic stress conditions, particularly dehydration in rice and other crop plants. It also elucidates the role of *RPL23A* in dehydration related stresses.

## Materials and methods

### Nucleotide sequence retrieval

The sequences of all the ribosomal protein-encoding genes of rice (RPL and RPS) were retrieved from RGAP-DB. The sequence identification and validation in RAP-DB, NCBI, and some other databases were carried out as described in Moin et al. (2016a,b) and Saha et al. (2017) to ensure their specificity. Primers were designed specifically for each of the identified genes using primer3 tool for studying their expression in response to different water stress related treatments.

### Dehydration treatments and sample collection for RP gene expression studies

To examine the responses of RP genes to different water stress treatments under greenhouse conditions (32 ± 2°C with relative humidity of 55 ± 5%), 1-month-old rice plants were subjected to limited water and drought stress treatments. The rice seeds of the variety, BPT-5204 were dehulled and surface sterilized with 70% ethanol for 1 min followed by the treatment with 4% sodium hypochlorite twice for 10 min. Seeds were then washed with sterile double-distilled water for 3–5 times, blot-dried and germinated on MS medium in the growth room (28 ± 2°C) for 15 days. After this, they were transferred to pots containing black alluvial soil in the greenhouse where they were further allowed to grow for 1 month. The 1-month-old WT rice plants were subjected to three different watering conditions; one set of plants were provided with ample amounts of water, about 500 ml/d as required for normal growth of rice. The other set was maintained under limited water conditions by providing minimal water (150 ml/day) so as to maintain barely moist conditions in the soil. There was no overlay or additional water in the pots of these plants. The third set of plants were allowed to grow under drought conditions without any water at all. These conditions were maintained up to 3 weeks (21 days). Before the initiation of drought stress treatments, a trial experiment was conducted to check the time that takes for the complete wilting of the majority of rice plants after withholding water from the pots. It was observed that more than 50% of rice plants became wilted between 15 and 21 days. Hence, 21 days treatment was considered as the permanent wilting point (PWP) for investigations on gene expression studies under drought stress. Drought stress induced to plant are usually represented in percent Field Capacity (FC), which mainly depends on the soil type. According to India water portal (http://www.indiawaterportal.org) for alluvial soils available in South India, which has been used in the current study for rice cultivation, the PWP occurs between 10 and 18% FC. In the present experiment, PWP was noticed on 21 day drought response, accordingly the FC on 3, 7, 15, and 21 day was ~60, 40, 20, and 15%, respectively. The leaf and root tissue samples were collected separately after 3, 7, 15, and 21 days treatment from each experimental sample in biological triplicates. The WT plants grown under well-watered conditions were considered as a control to normalize the expression of the corresponding treated samples.

A comprehensive expression analysis of all the RP genes that are involved in the assembly of both large and small subunits of rice (RPL and RPS) was performed using qRT-PCR. Total RNA was extracted from the root and shoot tissue samples separately in three biological replicates. About 100 mg samples of homogenized leaf and root tissues were transferred to sterile, DEPC (Diethyl Pyrocarbonate)-treated Eppendorf tubes containing 1 ml Trizol solution (Sigma-Aldrich, US) and centrifuged at 12,000 rpm at 4°C for 10 min. The supernatant was transferred to a fresh vial and 200 μl of chloroform was added. After a gentle mix and incubation for 5 min, the vial was centrifuged at 12,000 rpm at 4°C for 15 min. This step produced three phases, a red organic phase (containing proteins), an interphase (containing DNA) and a clear aqueous phase that contains RNA. This RNA containing phase was transferred to a fresh tube and 500 μl of ice cold isopropanol was added and allowed to stand for 10 min, after which the vial was centrifuged at 12,000 rpm at 4°C for 10 min. The supernatant was discarded and the clear RNA pellet was washed with 75% ethanol prepared in DEPC-treated water and centrifuged at 7,500 rpm at 4°C for 5 min. The pellet was air dried and dissolved in 20 μl DEPC-treated water. To avoid DNA contamination, total RNA was treated with RNase free DNase1 (Sigma-Aldrich, USA). The quality and quantity of RNA was checked using a spectrophotometer (NanoDrop Technologies Inc., USA) at A260/230 and A260/280 nm wavelengths. All the steps were carried out at 4°C and the vials involved in RNA isolation were treated with DEPC before use.

The cDNA was synthesized using reverse transcriptase (Takara, Clonetech, USA) and diluted in 1:7 proportion. The quantitative real time PCR (qRT-PCR) was performed to analyze the transcript levels of RPL and RPS genes in leaf and root tissues of rice plants grown at different levels of water as per Moin et al. (2016a). Tissues from plants grown with adequate water supply were used as control to normalize the corresponding fold change in gene expression. The qRT-PCR was performed using SYBR Green® Premix (Takara Bio, USA). The qRT-PCR cyclic conditions included an initial denaturation at 94°C for 2 min, followed by 40 cycles of 94°C for 15 s, an annealing temperature according to each gene for 25 s and 72°C for 30 s followed by a melting curve. The qRT-PCR was performed as three technical and biological repeats and the fold change was calculated using the ΔΔC_T_ method (Livak and Schmittgen, 2001).

### *RPL23A* construct preparation

The full-length cDNA sequence of *RPL23A* (460 bp) was retrieved from RGAP-DB. The sequence was also validated in RAP-DB and NCBI databases. When the *RPL23A* sequence from all the databases showed perfect match, the primers were designed according to the sequence with *Nco*I and *Xba*I restriction sites at the forward and reverse ends, respectively for subsequent cloning steps. The sequence was amplified from WT rice cDNA. The pRT100 (Addgene, A05521) was used as an intermediate vector to release the 35S promoter and poly-A tail along with the gene using *Nco*I and *Xba*I sites. The expression cassette of *RPL23A* was then cloned into the binary vector, pCAMBIA1300 using *Pst*I digestion. The vector carrying expression cassette of *RPL23A* was mobilized into *Agrobacterium tumefaciens* strain, EHA-105 for plant transformation.

### Plant growth conditions, *in planta* transformation of 35S: *RPL23A* in *indica* rice and selection of transgenic plants

The same variety of rice, BPT-5204 (Samba Mahsuri) that has been used in the expression studies was also used in generating the transgenic plants. The binary vector pCAMBIA1300 carrying full-length expression cassette of *RPL23A* was transformed into rice using *Agrobacterium*-mediated *in planta* transformation. The *in planta* transformation was performed according to the protocol described previously (Moin et al., 2016a). The transformation efficiency of the transgenic plants was almost the same as reported earlier (20%). After infection of the plants with *Agrobacterium* in the T_0_ generation, they were transferred to pots containing alluvial soil and maintained at 30 ± 2°C with 16 h of light followed by 8 h dark photoperiod. The T-DNA of the binary vector carries *hpt*II, as a resistance marker for the selection of transgenic plants in subsequent generations using the antibiotic, Hygromycin.

The seeds obtained from the *Agrobacterium*-treated plants (T_0_) were allowed to germinate on MS selection medium containing the antibiotic, Hygromycin (50 mgl^−1^). The seedlings that arose from the seed germinated on the selection medium were allowed to grow in the greenhouse and were further confirmed by PCR analysis of various elements present on the T-DNA of the binary vector. The plasmid of pCAMBIA1300 carrying *RPL23A* expression cassette was used as a Positive Control (PC), whereas the plants that were rescued from non-germinated seeds on selection medium followed by recovery on the selection-free medium were used as Negative Control (NC), and were represented as Null Segregant (NS) plants.

### Genomic DNA isolation and southern-blot hybridization

The genomic DNA was isolated using leaf tissue from T_2_ generation transgenic plants using the CTAB method with certain modifications. About 150 mg of leaf samples were used for grinding. To this, 1 ml of CTAB buffer + 20 μl β-mercapto-ethanol were added and the macerated tissue was incubated at 65°C. After 1 h, samples were centrifuged at 11,000 rpm for 15 min. To the supernatant that was transferred to a fresh vial, an equal volume of phenol: chloroform: iso-amyl alcohol (25:24:1) was added and the mixture was incubated at 4°C for 5 min followed by centrifugation at 5,000 rpm for 8 min. Incubation at 4°C (instead of the usual incubation at room temperature) resulted in better separation of proteins and nucleic-acids. An equal volume of chloroform: iso-amyl alcohol (24:1) was then added to the supernatant, which was further incubated at 4°C for 15 min with gentle shaking followed by centrifugation at 12,000 rpm for 12 min. This step was repeated twice. The clear upper phase was taken and an equal volume of iso-propanol was added and incubated for 8–12 h at −20°C. Genomic DNA was pelleted down, washed with 70% ethanol, air dried and dissolved in 100 μl nuclease free water. This resulted in the extraction of high-yield (2,000 ng μl^−1^) and good quality genomic DNA, which was free from protein and salt contamination (260/280 = 1.8, 260/230 = 2.1) and used in Southern analysis without the requirement for further purification.

Southern-blot hybridization was performed to confirm the transgenic nature of plants and also to determine the number of copies of T-DNA integration present in the genomes of the transgenic plants. Southern-blot hybridization was performed using 15 μg of high-quality genomic DNA. The genomic DNA was digested with *Sph*I restriction enzyme, whose site is absent in the T-DNA of the *RPL23A* overexpression vector and incubated at 37°C overnight. The PCR-amplified, DIG-dUTP (Roche, Germany) labeled fragment of the Hygromycin resistance gene (*hpt*II) was used as a probe in Southern hybridization analysis. Since *RPL23A*, which was used for overexpression studies in the present report is endogenous to rice, *hpt*II was used as a probe instead of *RPL23A*. This was to avoid the multiple banding patterns that might arise with the use of endogenous *RPL23A* (which also has gene copies) even in wild-type rice. The probe binding was detected with anti-DIG-alkaline phosphatase enzyme and NBT/BCIP substrate.

### Total RNA extraction, cDNA synthesis, semi-quantitative (Semi-Q) and quantitative real-time PCR (qRT-PCR)

Total RNA was isolated from the leaves and roots of 1-month-old *RPL23A* transgenic and NS plants using Trizol (Sigma-Aldrich, US) method as described earlier. The first strand cDNA was synthesized from 2 μg of total RNA using reverse transcriptase (Takara Bio, Clontech, USA). About 2 μl of 1:7 diluted cDNA was used for analyzing the *RPL23A* gene transcript levels in the selected transgenic lines. The rice *RPL23A* specific primers, designed through primer-3 tool were used in semi-Q and qRT-PCR. The cycle conditions for semi-Q PCR included an initial denaturation at 94°C for 3 min, followed by 26–28 repeated cycles of the 94°C for 30 s, 55°C for 25 s and 72°C for 30 s. This was followed by a final extension for 5 min at 72°C. The rice Actin, *act1* and tubulin, β-*tub* were used as endogenous control genes in qRT-PCR analyses for normalization.

Semi-Q and qRT-PCR analyses were performed on T_2_ generation rice transgenic plants to separate low and high expression lines of *RPL23A* transgenic rice plants. In the semi-Q analysis, the transcript levels of *RPL23A* in transgenic lines was measured based on the band intensity observed on the 1.5% agarose gel compared with the NS. The transgenic lines with weak and increased band intensity compared with the band intensities of NC were categorized as low and high expression lines, respectively. The transcript levels in transgenic lines were further validated through qRT-PCR. The same cDNA that was diluted in 1:7 proportions used in semi-Q PCR was also used in qRT-PCR to analyze the transcript levels in two classes of *RPL23A* lines (low and high) that were separated through semi-Q PCR. The cDNA synthesized from null or non-germinated seedlings was used as a Negative Control or Null Segregant (NC or NS) to normalize the expression pattern in qRT-PCR. The qRT-PCR reaction was performed using SYBR master mix (Takara Bio, USA) and the reaction conditions were similar as described above with an annealing temperature specific to *RPL23A* gene (56°C). The qRT-PCR data were analyzed according to the ΔΔC_T_ method (Livak and Schmittgen, 2001).

### Screening of *RPL23A* transgenic plants for water-use efficiency

In our previous report, we have already shown that the enhanced expression of *RPL23A* by the integrated 35S tetrameric enhancers in one stable *Ds* line among the activation tagged rice population resulted in enhanced WUE (Moin et al., 2016a). In the present study, we have overexpressed *RPL23A* and generated independent transgenic plants to validate the previous findings on the enhancement of WUE in rice by the activated expression of *RPL23A*. The two high expression lines along with NS were screened for WUE. After selection on Hygromycin (50 mg l^−1^) medium, the transgenic plants along with NS were transferred to black alluvial soil in the pots and provided ample amounts of water (up to 500 ml/day) for the first 4 weeks of transfer. After this, the overlaid water was removed from the pots and watering was restricted to about 150 ml/day so that only moist conditions were maintained in the soil. Limited water treatments were given to the rice plants as described earlier (Moin et al., 2016a). The phenotypic observations on the confirmed *RPL23A* transgenic plants in the T_3_ generation were performed in comparison with NS. The various phenotypic characters measured included the total number of tillers, productive tillers (tillers with panicles), panicle length and plant height. The data of all phenotypic parameters were collected from five individual plants of each transgenic line and NS.

### Δ^13^C analysis for water-use efficiency

Atmospheric Carbon exist as two isotopes, ^13^C and ^12^C with a molar ratio of 1:99. The diffusion of Carbon through stomata and assimilation by Ribulose-1,5-bis carboxylase/ oxygenase (RuBisCo) during photosynthesis discriminates between the two isotopes. During limited water supply, stomatal aperture tends to close to reduce water loss through transpiration resulting in a decrease in the concentration of intercellular CO_2_ (Ci). This discrimination by RuBisCo between the Carbon isotopes is high when internal CO_2_ is high and decreases with a decrease in Ci. Thus, the Δ^13^C value, which is the relative ratio of ^13^C/^12^C, expressed relative to the PDB standard, of a plant tissue reflects the capacity of a plant for gaseous exchange through stomata, integral Ci and overall WUE of a plant (Martin and Thorstenson, 1988; Farquhar et al., 1989; Bassett, 2013). The Δ^13^C is inversely related to WUE; lesser the Δ^13^C, higher will be WUE. The Δ^13^C value was measured using 500 mg of mature leaf samples collected from NS and four *RPL23A* T_1_ generation transgenic plants (parental plants, which were subsequently confirmed by Southern-blot analysis) after 1-month of growth under limited water conditions. Samples were dried at 65°C for 3 day in a hot-air oven, finely powdered and carbon isotope ratios were analyzed using an Isotope Ratio Mass Spectrometer (IRMS).

### Chlorophyll fluorescence

Chlorophyll fluorescence is a measure of the activity of photosystem II (PSII). It is also an indicator of plant response to environmental stresses and has been used to assess the overall photosynthetic performance of a plant (Murchie and Lawson, 2013). The chlorophyll fluorescence of *RPL23A* transgenic plants in the T_2_ generation was measured using a portable instrument, MINI-PAM essentially according to the manufacturer's protocol (Walz, Effeltrich, Germany; Murchie and Lawson, 2013; Batra et al., 2014). Readings were taken in the two high expression lines, just before the treatment (1 month after transfer to pots) and 1 month after the start of limited water conditions. Each set of readings was taken in triplicates and the quantum efficiency (*F*_*v*_/*F*_*m*_) of transgenic plants was compared with the NS plants and represented in the form of bar diagrams.

### Expression studies of RP genes in *RPL23A* high expression lines

The two high expression lines of *RPL23A* were selected to check the transcript levels of other RP genes to gain insights into whether they are differentially regulated by *RPL23A*. For this qRT-PCR was performed using primers of 70 RP genes in two selected high expression lines. The fold change was calculated using ΔΔC_T_ and the expression was normalized with an NS plant that was used in the separation of high and low expression lines.

### Stress assays at seedling stage and measurement of growth parameters

After 7 day of germination, the transgenic and NS seedlings were transferred to test tubes. To evaluate the response of *RPL23A* transgenic lines to NaCl and simulated drought stress (other than WUE), the T_3_ generation seedlings were allowed to grow in the solutions of NaCl (100 mM) and PEG-6000 (10%) prepared in distilled water for 10 day. The corresponding seedlings grown in water (without a stress-inducing agent) were considered as untreated or control. The fresh weight (FW) and root lengths (RL) of two high expression lines were measured using a scale bar after 10 day of treatment with PEG and NaCl with respect to NS plants. The FW and RL were measured in triplicates and the data were represented in the form of bar diagrams.

### Estimation of chlorophyll and proline contents

The osmoprotectant, proline was estimated in two high expression lines and corresponding NS plants at the T_3_ generation seedling stage. The two high expression and NS seedlings, after an initial germination for 1 week were allowed to grow in salt (100 mM), PEG (10%) and water for 10 days. About 100 mg of leaf-derived tissue from 10 day old treated and untreated seedlings was homogenized in 5 ml of 3% aqueous sulfosalicylic acid. The leaf homogenate was then centrifuged at 12,000 rpm for 15 min. Then, 400 μl of supernatant was mixed with equal volumes of 400 μl acid ninhydrin and glacial acetic acid and incubated for 1 h at 100°C. The reaction mixture was then mixed with 800 μl toluene. The organic phase was used for measuring absorbance at 520 nm wavelength using toluene as a blank. Proline concentration was measured from the standard curve using the method described by Bates et al. (1973).

The chlorophyll content was also measured in the seedling stage of T_3_ generation transgenic and NS plants. The chlorophyll was extracted using 100 mg of leaf tissues in 80% acetone and absorption of the extracts was measured at OD 663 nm and 645 nm using a UV spectrophotometer (UV-1800 Shimadzu) as per the established protocols (Arnon, 1949; Zhang et al., 2009). The samples used for chlorophyll estimation included leaves of 10 day old transgenic and NS seedlings that were grown in NaCl (100 mM), PEG (10%) and water collected in triplicates to measure the concentrations of Chl-a, Chl-b, and total chlorophyll (Chl-t).

### Expression studies of stress-specific genes in *RPL23A* transgenics

The transcript levels of six stress-specific genes, such as *bZIP23, WRKY72, DREB2B, LEA3-1, SNAC1*, and *SNAC2* were studied in high expression lines of *RPL23A*. These genes were selected as they have been reported to be to be involved in conferring tolerance to different abiotic stresses, particularly drought and salt in rice. This analysis was performed in the shoot and root tissues of PEG-treated transgenic seedlings for 48 h, and the expression data was normalized with corresponding untreated transgenic samples.

### Statistical analysis

The qRT-PCR, phenotypic observations (tillering and seed yield) and physiological experiments (fresh weight, root length and proline and chlorophyll contents) were carried out in three biological and three technical replicates. The fold change in each qRT-PCR experiment in the present study is normalized with two rice specific reference genes, *act1* and β-*tub*. The mean fold change obtained after normalization with these two genes was considered as final fold change. The mean of the observations (qRT-PCR, phenotypic and physiological studies) was represented in the form of bar diagrams constructed using SigmaPlot v11. One-way ANOVA was used to study the statistical significance, and the significance was represented at *P* < 0.05 with asterisks in the graphs. The qRT-PCR data was also represented in the form of heat maps constructed using the mean of fold change obtained from biological and technical triplicates. Heat maps were developed using Morpheus program.

## Results

### Differential transcriptional regulation of RP genes under dehydration stress treatments

A keyword search of “ribosomal” resulted in the identification of 70 representative genes that are involved in the assembly of both large and small subunits (Moin et al., 2016a,b; Saha et al., 2017).

To gain insights into whether RP genes respond differentially to dehydration treatments at the greenhouse level, WT rice plants were subjected to different dehydration treatments and the expression profiles of both large and small subunit genes were analyzed periodically. After 21 days treatment, rice plants grown under drought conditions became completely dried, whereas those grown under limited water started to wilt (Supplementary Figure 1). The fold change obtained from dehydration treated samples was compared with corresponding control samples grown under normal conditions providing ample amounts of water.

All the RP genes exhibited differential expression in response to water deficit treatments in both shoot and root tissues. The majority of genes became upregulated at some time point or the other indicating that the majority of them respond to dehydration treatments positively. The genes that exhibited ≥2-fold transcript level on the log_2_ scale were considered as upregulated. In shoots, about 30 RPL genes (88%) became activated under limited water conditions after 3 days of treatment. Among these, 25 (73%), 12 (35%), and 11 (32%) RPL genes, respectively maintained the expression levels consistently up to 7, 15, and 21 days. In roots, initially 11 RPL genes (32%) became upregulated after the onset of limited water stress and as the treatment progressed, many other genes began to express. After 21 days, 25 RPL genes (65%) showed significant expression. About 75–80% of RPS genes became upregulated during 3–15 day treatment, of which some of them were downregulated as the treatment progressed. About 60% maintained continuously enhanced expression levels up to the last time period (21 days) in shoots. However, only 7 RPS genes were activated in roots on the 3rd day of treatment, but as the treatment progressed, 18 (52%), 27 (79%), and 29 (85%) genes became upregulated. Under drought conditions, 83% of RPL genes started expression in shoots and as the treatment continued, some of them became down-regulated. Around 76, 52, and 41% of RPL genes, respectively expressed at 17, 15, and 21 day time intervals. In roots, the number of RPL genes that expressed gradually increased from 44% on 3rd day to 52% on 7th day, 64% on 15 day, and 70% on the 21st day. The number of RPS genes expressed in shoots under drought stress ranged from 35 to 88%, while in roots, the number of upregulated RPS genes ranged from 20 to 80%.

To analyze the level of expression of 70 RP genes in detail, they were categorized as low, if the fold is 2–5; moderate, if they exhibit 5–10 fold; and significant, if the transcript levels were more than 10-fold. Among the genes that showed significant of expression in shoots at a given time period under both limited water and drought treatments included *RPL6, 7, 19, 21.2, 23A, 18, 26, 27, 28, 36, 37*, and *51*; and *RPS4, 5, 7a, 10, 17, 18a, 19, 20, 21*, and *23a*, whereas, *RPL6, 7, 10, 11, 13a, 13b, 18p, 21, 23A, 24, 26, 37*, and *44*; and *RPS9, 10, 18, 21, 23, 24, 25*, and *27* became significantly upregulated in roots in response to both the treatments.

In shoots, *RPL6, 7, 19-3, 21.2, 18A, 23A, 24, 31, 34, 35A, 37.1*, and *51*; *RPS4, 10, 18, 19, 23, 24, 26*, and *29* showed high expression (>10-fold) throughout the duration of dehydration treatments, in both limited water and drought conditions at all the time intervals (3, 7, 15, and 21 day) studied. Similarly, in roots, *RPL6, 7, 11, 13b, 18p, 23A, 24, 26.1, 30, 31, 32, 35a, 36.2; RPS4, 6, 6a, 9, 10, 13, 17, 18, 23, 25, 27, 28*, and *29* exhibited high levels of transcripts after the onset of the treatment until the last time point. *RPL6, 7, 23A, 24*, and *31; RPS4, 10* and *18* became upregulated in both shoot and root tissues (Supplementary Figures 2A–P). These genes can be considered to be promising in bringing about tolerance under water deficit treatments as they consistently became upregulated in both the tissues. This also clearly indicates that they have a common role in inducing tolerance to both water deficit and drought stress conditions. The overlap in up-regulation (Supplementary Figure 3A) and down-regulation (Supplementary Figure 3B) of RP genes in response to limited water and drought conditions in the shoot and root tissues separately were represented in the form of Venn diagrams. Supplementary Table 1 provides a detailed list of genes that exhibited overlap in the up-regulation (Supplementary Tables 1A–H) and down-regulation (Supplementary Tables 1I–P) in the shoot and root tissues at each time point.

**Figure 1:**
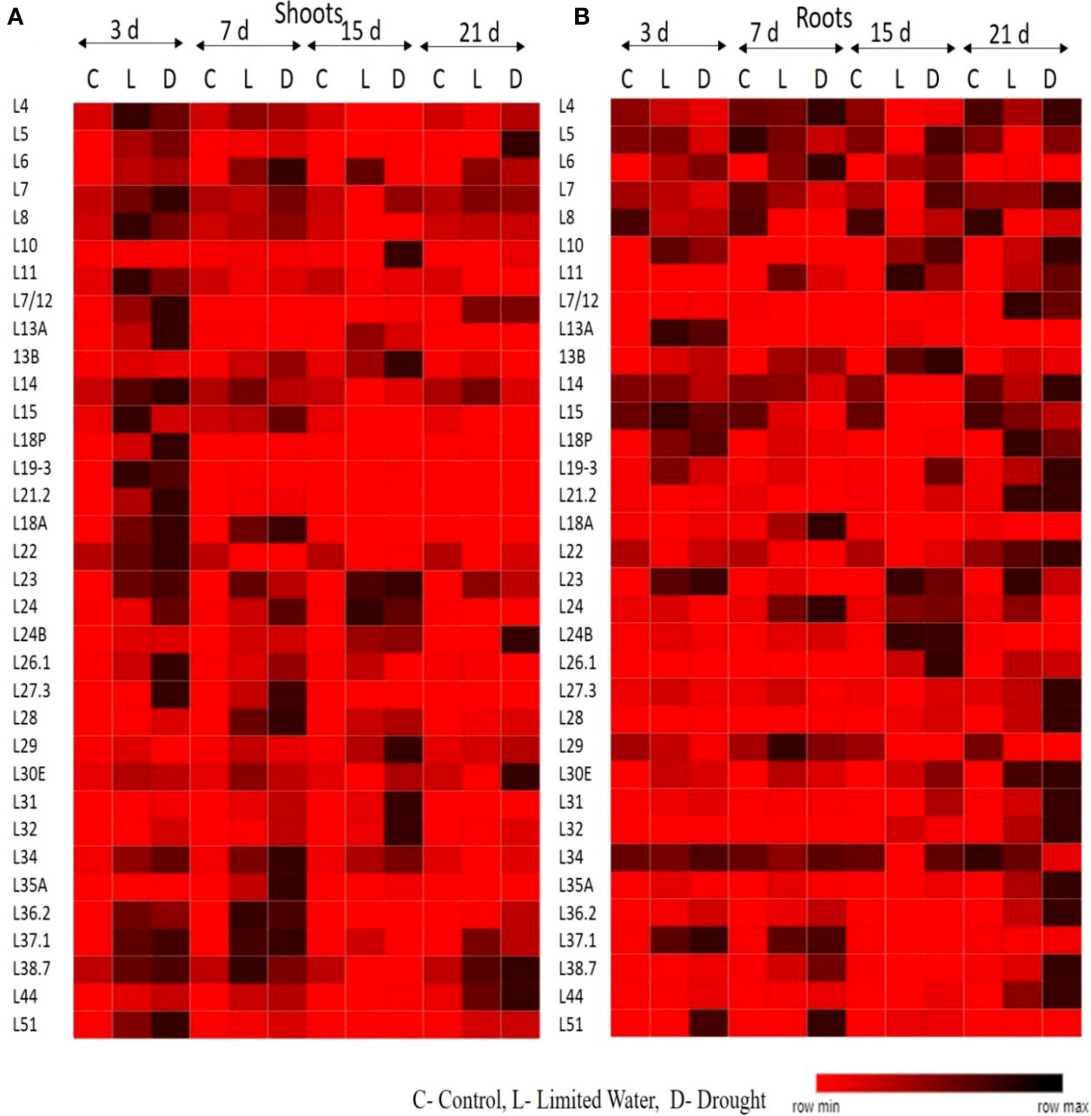
Expression pattern of RPL genes in response to limited water and drought conditions. One-month-old rice plants were exposed to different dehydration, such as limited water and drought at four different time points as indicated on the top. The qRT-PCR is used to determine the expression levels of RPL genes in **(A)** shoot and **(B)** root tissues and the fold change were normalized using ΔΔC_T_ method relative to that in untreated plants at corresponding time points. Rice actin (*act1*) and tubulin (β-*tub*) were used as internal reference genes for normalization of fold change. Three biological replicates and three technical replicates were included in the study. The light colored grids in the heat maps represent the weak expression, while the dark-colored grids indicate significant expression.

**Figure 2:**
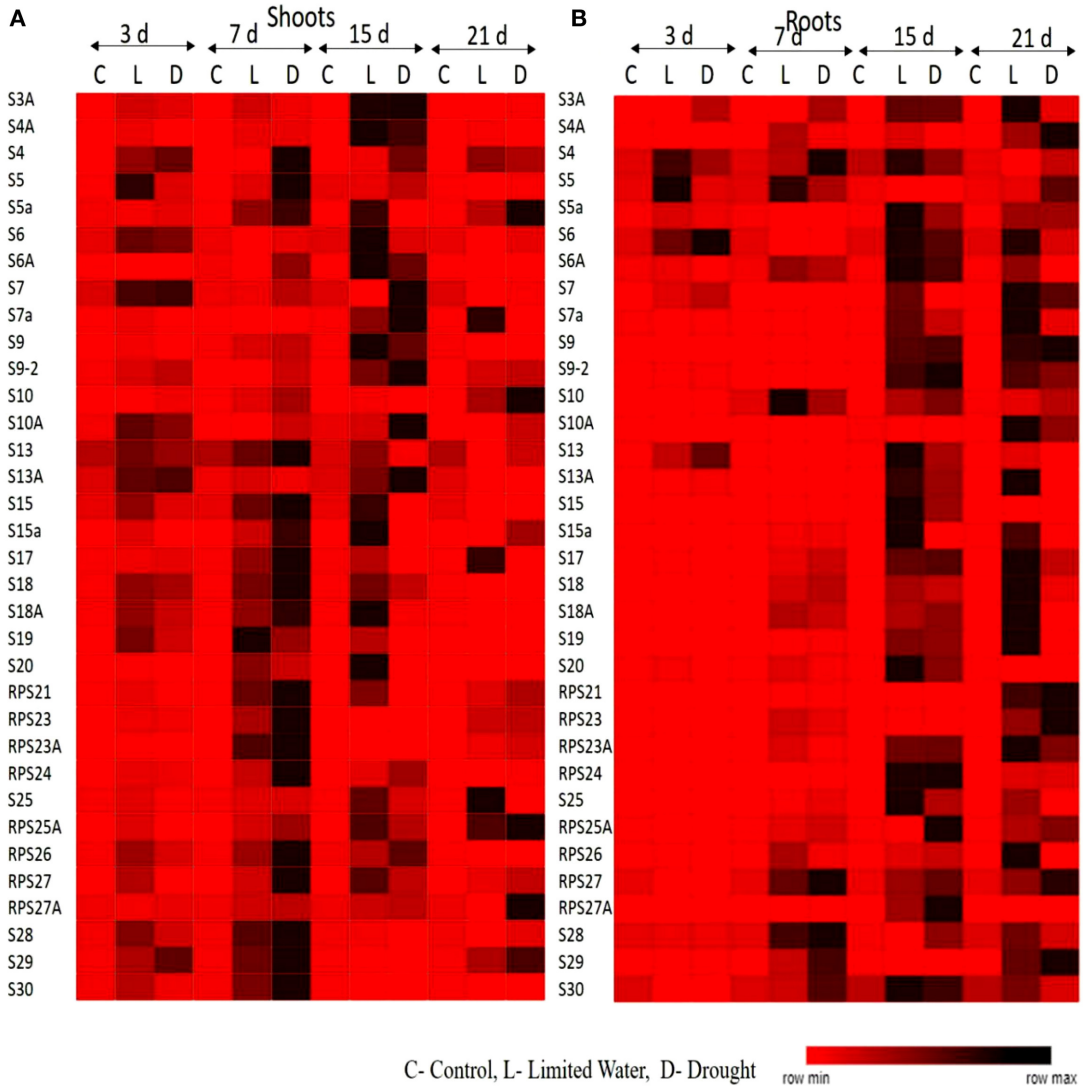
Heat map representation of expression pattern of RPS genes in response to limited water and drought conditions. The expression pattern of RPS genes in **(A)** shoot and **(B)** root tissues were represented in the form of heat maps. The final fold change was normalized using ΔΔC_T_ method with two reference genes (*act1* and β-*tub*).

The upregulation of these RPL genes is in accordance with our previous observations, where we have studied the expression of RPL genes in treatments with stress-inducing agents and at different developmental stages of the cultivar BPT-5204, which is also the test material in the present study (Moin et al., 2016b). The expression patterns of RPL (Figure 1) and RPS (Figure 2) genes have been represented in the form of heat maps, which were generated using the mean of fold change obtained from different biological and technical triplicates. The dark-colored grids in the heat maps indicate significantly enhanced expression, while light-colored grids represent weak expression.

### Screening and molecular investigations on *RPL23A* transgenic plants

**Figure 3:**
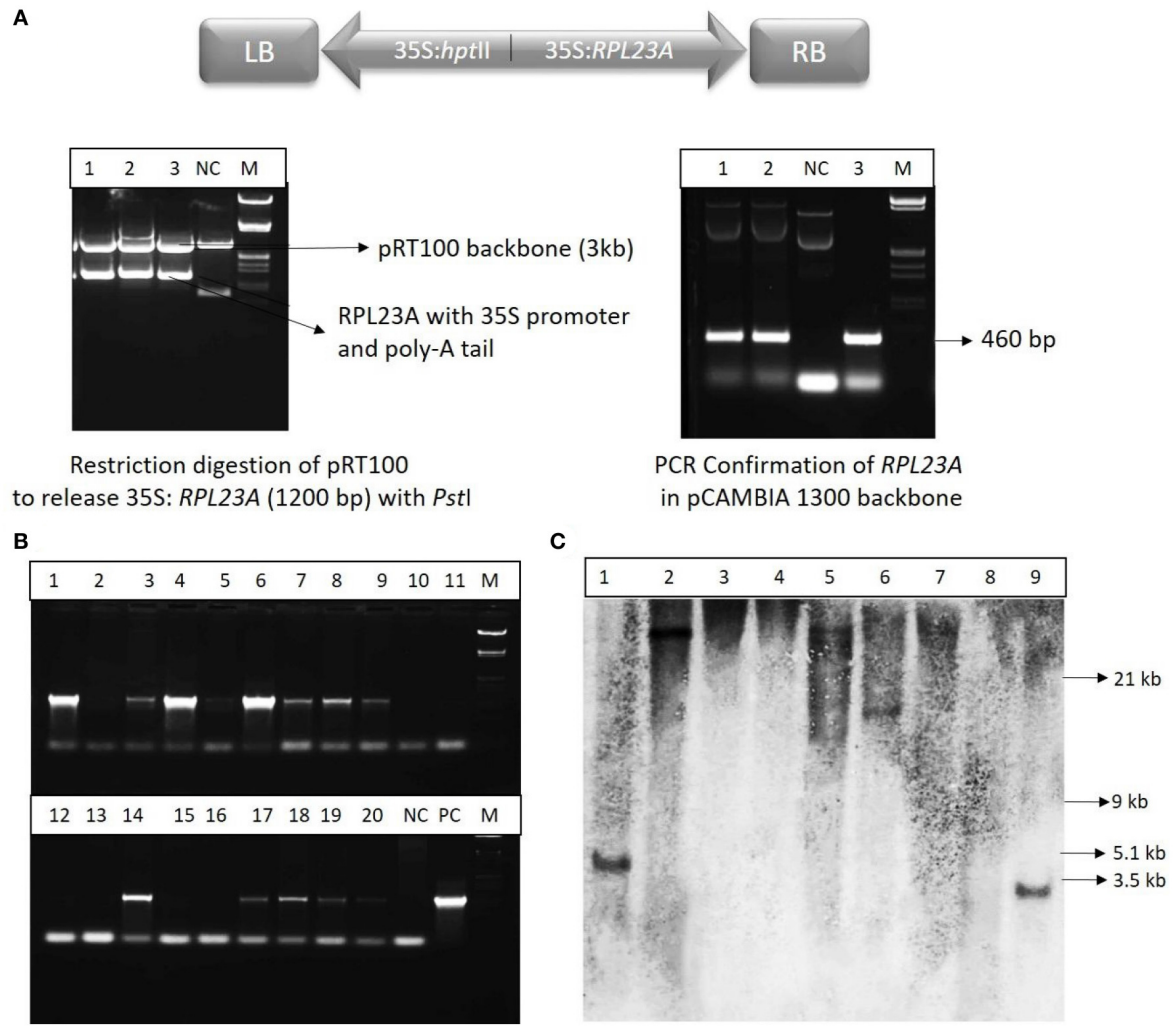
Cloning and molecular investigations of *RPL23A*. **(A)** Map of T-DNA of the binary vector, pCAMBIA1300 carrying expression cassettes of *RPL23A* and *hpt*II. RB and LB, Right and Left borders of the T-DNA, respectively; 35S, CaMV35S promoter. The *RPL23A* was initially cloned into the intermediate vector, pRT100 to release the gene along with the expression cassette. (Left) The pRT100-*RPL23A* clone was digested with a *Pst*I restriction enzyme to release the 1,200 bp *RPL23A* cassette. (Right) After cloning of the cassette in pCAMBIA1300, it was further confirmed by PCR that amplifies the gene (460 bp). NC, Negative Control; PC, Positive Control and M, λ *EcoR*I*-Hind*III marker. After transformation, the transgenic plants were confirmed by **(B)** PCR and **(C)** Southern-blot hybridization. Lanes 1-20 (in PCR) and 1-9 (in Southern-blot) refers to transgenic samples. NC, Negative Control; PC, Positive Control. The sizes labeled in Southern-blot are according to λ *EcoR*I*-Hind*III marker.

The *RPL23A* construct (Figure 3A) was confirmed by restriction digestion using *Pst*I enzyme, which released the *RPL23A* expression cassette of ~1,200 bp (Figure 3B). The binary vector was also confirmed by PCR analysis. The seeds obtained from the primary generation *Agrobacterium*-treated plants (T_0_) were screened on Hygromycin selection medium (50 mg l^−1^) as the binary vector contains *hpt*II as a selection marker that confers resistance to the antibiotic, Hygromycin. The putative transgenic seedlings continued further growth within 4–5 day after inoculation, while non-transgenic and WT seeds became bleached. After selection, plants were analyzed by PCR amplification using *hpt*II gene in the T-DNA using specific primers (Figure 3C). Since the WT and NS plants also contain *RPL23A* in their genome, *hpt*II was used for PCR analysis, but not *RPL23A*. About 400 seeds were infected with *Agrobacterium* carrying the binary vector, pCAMBIA-*RPL23A*, from these 95 (23%) were found to be positive through antibiotic selection and PCR amplification. The *hpt*II resistant and PCR positive plants obtained from selfing of the *Agrobacterium*-treated plants were selected for developing homozygous lines and Southern-blot hybridization. Further, among the positive transgenic plants, only those that were confirmed through Southern-blot hybridization were selected for detailed molecular investigations.

Some of the transgenic plants that were positive for antibiotic selection and PCR amplification were selected for Southern-blot hybridization analysis. The independent nature of T-DNA integration into the genome of transgenic plants was identified by the different restriction fragments of the transgenic plants binding to the probe. Of the nine samples analyzed, single insertions were found in five lines. Of these five lines, three were high expression and two were low expression lines. Although there were many high and low expression lines observed through semi-Q and qRT-PCR, only those that were confirmed through Southern-blot hybridization were selected for further molecular investigations.

**Figure 4:**
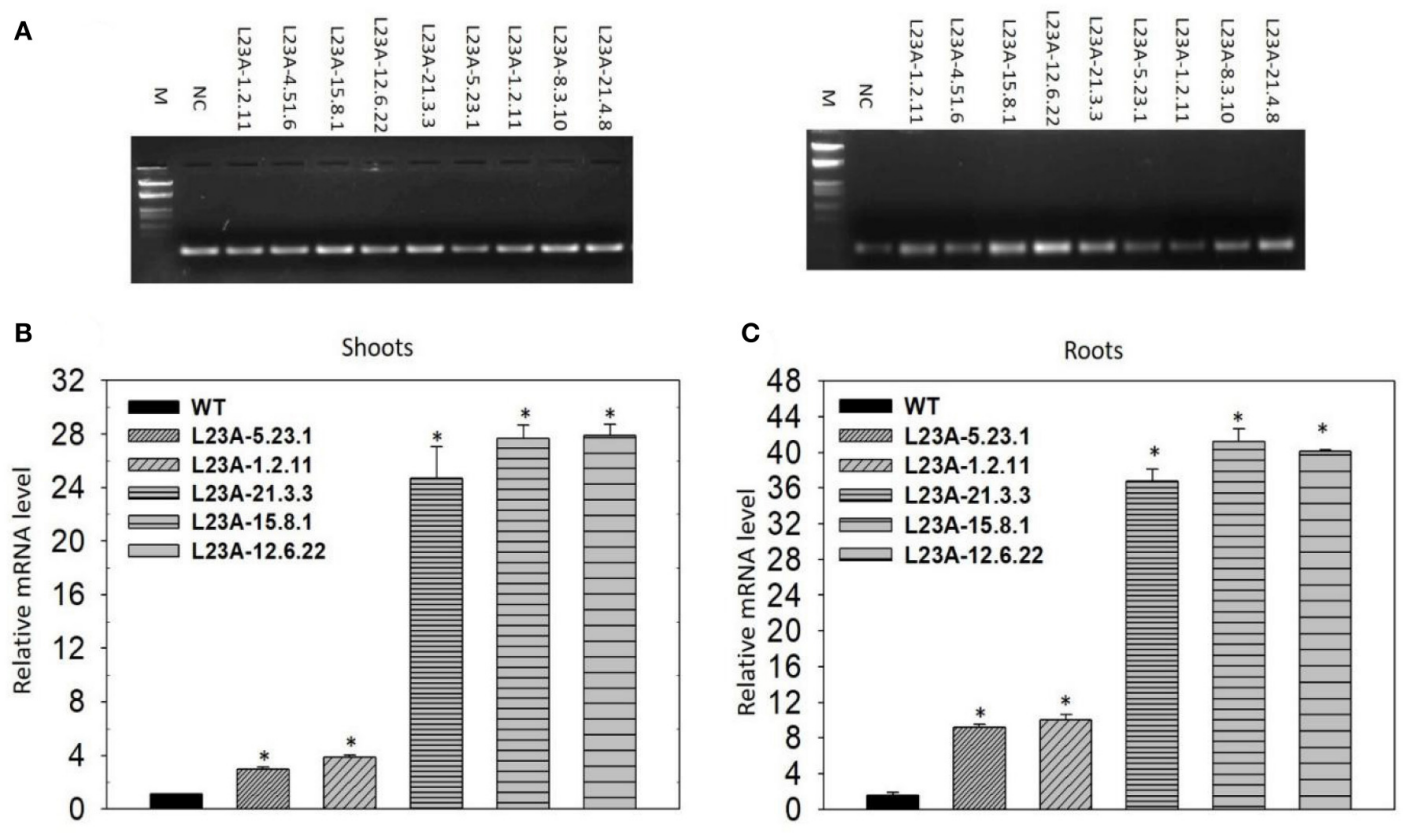
Semi-quantitative and quantitate PCR analysis of low and high expression lines. **(A)** (Left) Rice actin (*act1*) was used as an internal reference gene. (Right) *RPL23A* was used to assess the transcript levels to separate low and high expression levels in comparison with the Null Segregant (NS), also called Negative Control (NC). Based on the band intensity on the gel, lines L23A-15.8.1, L23A-12.6.22, and L23A-21.3.3 considered as high expression lines and the remaining lines were considered as low expression lines. The results on semi-Q were validated by qRT-PCR in **(B)** shoot and **(C)** root tissues, which also resulted in similar observations. The high expression lines exhibited >25-fold change in shoots and roots, while low expression lines had <5-fold change in both the tissues. This expression analysis was performed in the T_2_ generation transgenic plants. NC was used to normalize the expression pattern of *RPL23A* in transgenic plants. The relative expression was considered statistically significant at *P* < 0.05 which is represented with asterisks in the graph based on one-way ANOVA.

The T_2_ generation rice transgenic plants were separated into low and high expression lines based on the band intensity of *RPL23A* transcripts observed through semi-Q PCR on an agarose gel. Rice *actin* was used to normalize the expression patterns (Figure 4A). Based on the band intensity, lines were categorized into low and high expression using primers specific to *RPL23A*. A total of ten transgenic plants were selected for expression analysis through semi-Q PCR. The transcript levels of some of them were further determined by qRT-PCR using the NS as a negative control to normalize the expression pattern of other low and high expression lines. The lines with transcript levels <5-fold were considered as low, while those with more than 5-fold increase in expression were categorized as high expression lines. Three lines, L23A-15.8.1, L23A-12.6.22, and L23A-21.3.3 were identified as high expression lines having transcript levels ranging from 25 to 30-fold in shoots (Figure 4B) and up to 45-fold in roots (Figure 4C). The two transgenic lines, L23A-5.23.1 and L23A-1.2.11 that were identified as low expression lines through semi-Q PCR were selected to compare their expression with high expression lines and it was observed that the transcript levels of L23A-5.23.1 and L23A-1.2.11 ranged between 2- and 5 fold in both shoots and roots corroborating the observations made through semi-Q PCR. The high expression lines, L23A-15.8.1 and L23A-12.6.22 with single gene insertion observed through Southern analysis were selected for all the physiological and quantitative studies and their experimental readings were compared with the NS plants.

### Transcriptional regulation of RP genes by *RPL23A*

**Figure 5:**
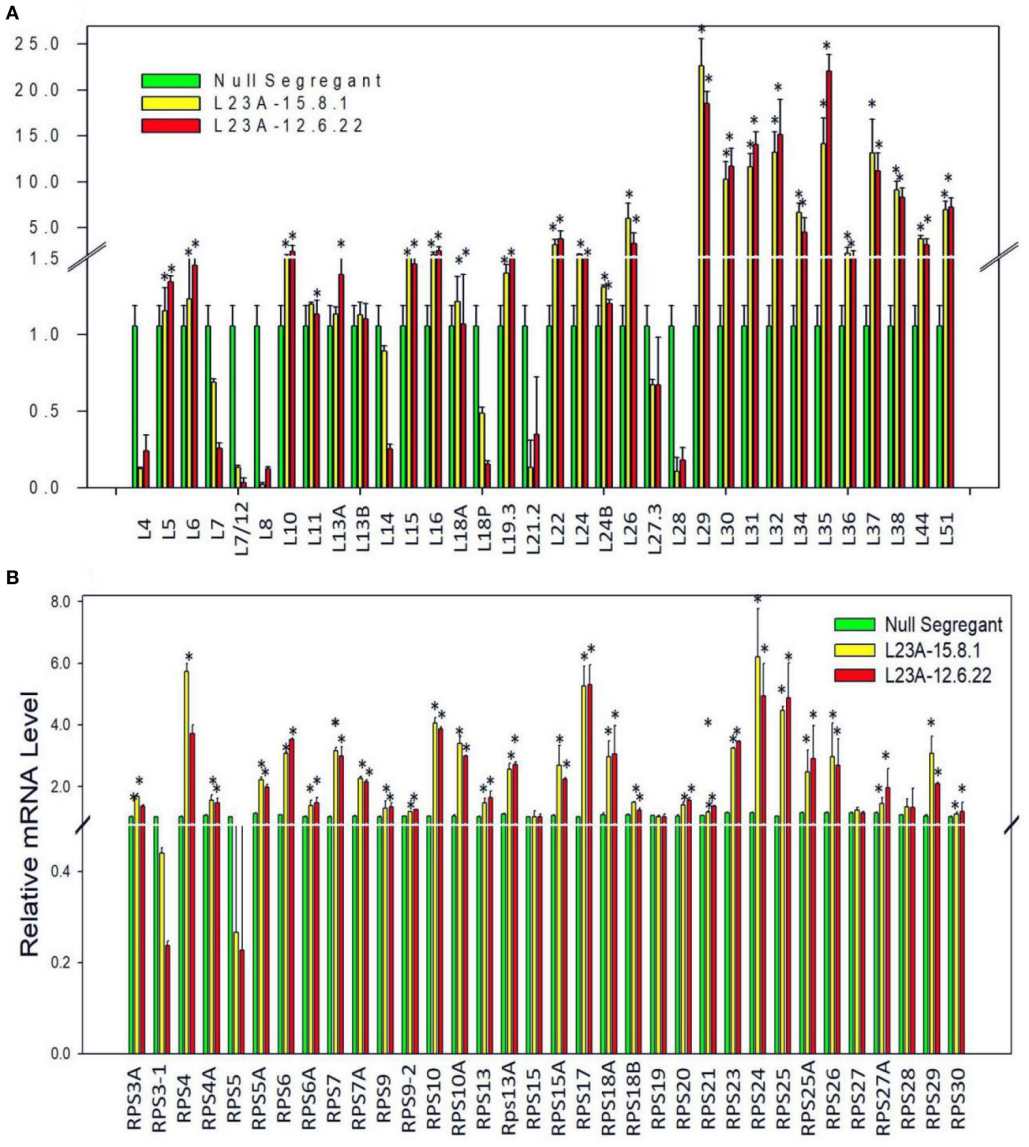
Expression analysis of RPL and RPS genes in high expression lines of *RPL23A*. The two high expression lines of *RPL23A* (15.8.1 and 12.6.22) in the T_2_ generation were used to check the expression levels of other members of RP gene family in order to understand whether overexpression of *RPL23A* activates other **(A)** RPL and **(B)** RPS genes as well. About 50% of both RPL and RPS family members showed an expression of >2-fold. RPL 29, 30, 31, 32, 35, 37, and 38, and RPS 4, 10, 17, 18a, 24, and 25 became significantly upregulated, possibly indicating that *RPL23A* functions in association with these genes. The statistical significance was calculated at *P* < 0.05 and is represented with asterisks in the graphs based on one-way ANOVA.

To check whether overexpression of *RPL23A* is associated with differential expression of other members of RP gene family, we have studied the expression levels of all the RP genes in two high expression lines of *RPL23A*. About 50% of both RPL and RPS family members showed an expression of more than 2-fold increase. *RPL29, 30, 31, 32, 35, 37*, and *38*, and *RPS4, 10, 17, 18a, 24*, and *25* became significantly upregulated indicating the possibility of a cross talk between *RPL23A* and these genes (Figure 5).

### Phenotypic and physiological characterization of transgenic plants

**Figure 6:**
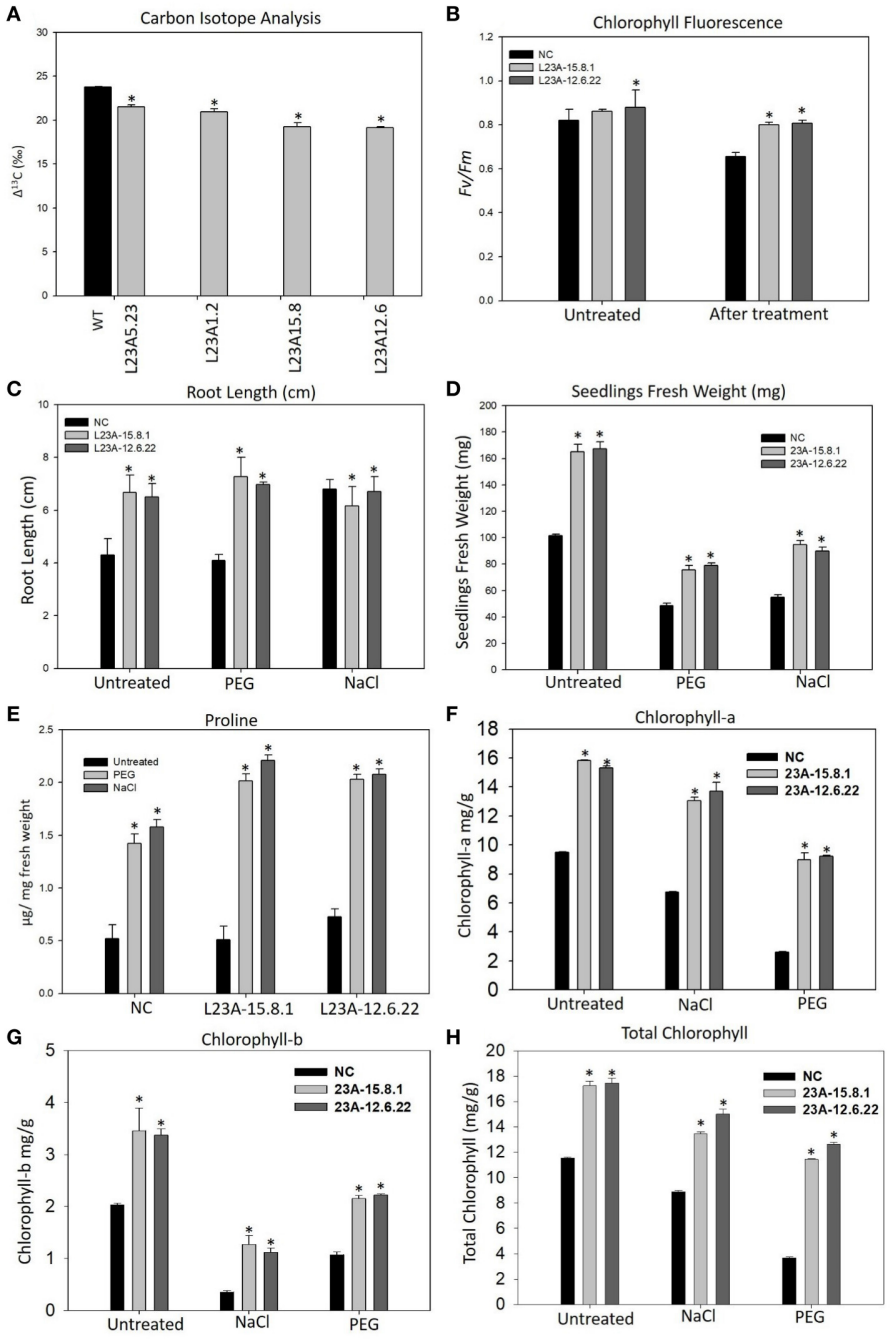
Physiological analysis of high expression lines of *RPL23A*. **(A)** The Δ^13^C measured in two high, L23A-15.8 and L23A-12.6, and two low expression, L23A-5.23, L23A-1.2 lines in T_1_ generation had values of 19.24, 19.14, 21.5, and 20.9‰, respectively with respect to NS which had 23.75‰. **(B)** The chlorophyll fluorescence of two selected lines in the T_2_ generation, performed with MINI-PAM had 0.86 and 0.88 under normal conditions, whereas under limited water conditions, both the lines showed *Fv/Fm* of 0.80. **(C,D)** The T_3_ transgenic seedlings showed increased growth parameters at seedling stage under normal and treated conditions with respect to NC. The primary root was considered to calculate the root length using a 1 cm scale bar. **(E)** Proline content of T_3_ transgenic and NC seedlings with and without PEG and NaCl treatments. **(F–H)** Chlorophyll measurements in the T_3_ generation transgenic seedlings. The seedlings exhibited high levels of **(A,B)** and total chlorophyll contents with respect to NC. Mean values of chlorophyll data with ± standard error represented with asterisks were considered statistically significant at *P* < 0.05.

The high expression lines of *RPL23A* showed increased yield-related parameters, such as tillering, panicle number and size and total seed yield under limited water conditions with respect to WT (Supplementary Table 2). The Δ^13^C measured in two high, L23A-15.8 and L23A-12.6, and two low expression, L23A-5.23, L23A-1.2 parental lines had values of 19.24, 19.14, 21.5, and 20.9‰, respectively with respect to NS, which had 23.75‰ (Figure 6A). The chlorophyll fluorescence of *RPL23A* transgenic plants in the T_2_ generation was measured using MINI-PAM. The two high expression lines, L23A-15.8.1 and L23A-12.6.22 showed a quantum efficiency (*Fv*/*Fm*) of 0.86 and 0.88, respectively just before the water withdrawal treatments, with respect to NS (0.82). Interestingly, 1 month after limited water availability (150 ml/day), both the lines had the quantum efficiency of 0.80, which was close to the well-watered conditions and significantly greater than the NS, whose quantum efficiency was only 0.65 (Figure 6B).

The transgenic seedlings exhibited better tolerance to simulated drought (10% of PEG-6000) and salt (100 mM NaCl) treatments (Supplementary Figure 4). The root length (RL) of NC and transgenic seedlings was 4 and 6.5, respectively under normal (untreated) conditions. In response to PEG, there was not much change in RL. However, the RL under NaCl treatment of untreated seedlings increased from 4 to 6 cm, which is equivalent to that of transgenic seedlings (Figure 6C). The untreated seedlings of high expression lines exhibited a total FW of 155–160 mg, which was 1.5 fold higher than the corresponding NS seedlings (100 mg). Under PEG, the high expression lines had an FW of 75–80 mg, while NC exhibited only 45 mg. Similarly, the FW of high expression lines in response to NaCl treatment was up to 100 mg, which is 2-fold greater than NS (50 mg) (Figure 6D).

The proline content was found to be increased in both transgenic and NS seedlings after treatment with PEG and NaCl. However, the proline in transgenic seedlings was 0.5–1 fold higher than NS (Figure 6E). The increase in proline content indicates that cytosolic osmotic potential is maintained in transgenic lines even under the conditions of stress.

The content of Chl-a in seedlings of NS and high expression lines, L23A-15.8.1 and L23A-12.6.22 under untreated condition was up to 9.5, 15.8, and 15.3 mg/g, respectively. After exposure to PEG and NaCl treatments, the level was slightly reduced to 13.04 and 13.7 mg/g in high expression lines but higher than NC (6.73 mg/g) (Figure 6F). The Chl-b content in high expression lines was around 3.5 mg/g under normal condition with respect to NS (2.02 mg/g), while in response to PEG and NaCl, Chl-b was slightly decreased but higher than NS (Figure 6G). The total chlorophyll content under normal conditions in high expression lines was around 17 mg/g compared with NC (11.5 mg/g). After treatment with NaCl and PEG, the Chl-t in high expression lines was in the range of 11–14 mg/g, which was significantly higher than NS under both the conditions (Figure 6H).

### Expression profiling of stress-specific genes

Overexpression studies of *LEA3-1, bZIP23, NAC*, and *DREB* genes in rice showed enhanced drought tolerance and yield (Chen et al., 2008; Xiang et al., 2008; Zheng et al., 2009; Liu et al., 2014). The transcription factors, such as *OsbZIP23, OsNAC1, OsNAC2*, and *OsWRKY72* have also been shown to be highly expressed by drought and salt stresses in rice. All these genes also became upregulated in the high expression lines of *RPL23A* under simulated drought treatment (Supplementary Figure 5). Particularly, the genes *bZIP23, DREB2B, LEA3-1*, and *SNAC-1* became several fold upregulated in the transgenic rice plants overexpressing RPL23A suggesting that it played an important role in the proper targeting of these stress-specific proteins under the conditions of stress.

## Discussion

Water deficiency affects differently at different stages of rice growth. Water stress at vegetative stages causes reduced plant height, reduced tillering and overall reduction in plant biomass. Significantly, the deficiency at reproductive stages results in the reduction of fertile panicle formation, percent grain filling and thereby a greater reduction in grain yield (Munns and Weir, 1981; Biswas and Choudhuri, 1984; Blum, 1988). If water-use efficient rice is developed through transgenic technology, an extensive amount of irrigation is saved, which could be used to increase the productivity of other water-demanding crops.

For the first time, we have reported the development of a sufficiently large enhancer-based activation tagged population in *indica* rice. The screening of these mutants for WUE revealed the activation of two RPL genes, *RPL6* and *RPL23A* by the integrated tetrameric 35S enhancers (Moin et al., 2016a). Further investigation of the entire RPL gene family also revealed their significant upregulation in abiotic and biotic stress responses in rice (Moin et al., 2016b). Similar enhancement in expression of genes coding for RPS has also been observed in another recent study (Saha et al., 2017). The Arabidopsis genome has a total of 247 RP genes that include 98 RPS genes and 143 RPL genes (Wang et al., 2013), whereas rice has a total of 57 RPS gene and 123 RPL gene copies (Moin et al., 2016b).

In the present study, we have demonstrated that a vast majority of RP genes became significantly upregulated at different stages of dehydration stress, including both limited water and drought stress. Among the 70 RP genes analyzed, a majority (>50%) of them were upregulated. *RPL6, 7, 23A, 24*, and *31* and *RPS4, 10* and *18* genes exhibited an overlap in the upregulation under both limited water and drought treatments in shoot and root tissues indicating that they might have a role in inducing tolerance. The up-regulation of these RPL and RPS genes are in agreement with the earlier reports (Kawasaki et al., 2001; Moin et al., 2016b; Saha et al., 2017).

The overlap in expression of *RPL6* and *RPL23A* at all the stages of dehydration treatment is also consistent with our previous findings (Moin et al., 2016a). The significant and immediate upregulation of these genes after the onset of stress might be a cellular necessity to maintain the integrity and stability of the ribosomal complex so that the translation of other proteins is not hampered thereby conferring an early defense to the plant against the impending stress. *RPL23A* has been physically mapped near the polypeptide exit tunnel of the ribosomal complex (Maier et al., 2005). This position suggests a role for *RPL23A* in protein translocation and secretion, which has been validated both in prokaryotes and eukaryotes (Halic et al., 2004; Maier et al., 2005; Menetret et al., 2005). Hence, upregulation and overexpression of this gene might ensure that the process of protein secretion occurs flawlessly, particularly under stress. In other words, this process assures that all the important proteins of the cell, such as transcription factors, kinases, membrane channels, repair proteins and so on are precisely targeted even under the conditions of stress. The several fold upregulation of some important stress-specific genes like *bZIP23, LEA3-1, SNAC1*, and *DREB2B* in high expression lines of *RPL23A* also indicates that the synthesis and targeting of these proteins occurred possibly more efficiently under stress. Therefore, the stress-tolerant properties of *RPL23A* as observed in our studies might have probably emanated from its site of location in the ribosomal complex.

The previous reports suggested that RPL and RPS genes have roles not only in growth and development but also in abiotic and biotic stress responsiveness and tolerance. RPL10, which is involved in combining the 40 and 60S subunits of the ribosome is an important protein in the formation of the functional 80S ribosome (Eisinger et al., 1997). It also provides the interaction sites for aminoacyl-tRNA during translation (Hofer et al., 2007). The Maize and Arabidopsis *RPL10* genes had been reported for their significant upregulation in shoots and roots upon exposure to UV-B radiation (Ferreyra et al., 2010). In the present study also, *RPL10* was highly upregulated in shoots and roots under both limited water and drought treatments. Significant expression of RPL10 in response to multiple stress treatments suggests its important role in maintaining the 80S ribosomal function and also in the regulation of translational activities in the cell under stress conditions. Similarly, RPL19, a component of the large subunit of ribosome interacts with L14 and L3 and also with rRNAs of the large subunit to maintain the stability of ribosomes (Harms et al., 2001). The *Nicotiana benthamiana* NbRPL19 had been reported in the calmodulin-mediated regulation of protein synthesis during carbon assimilation (Mönke and Sonnewald, 1995). Also, RPL19 has RNA-chaperone activity and is involved in Thymidylate Synthase gene splicing (Semrad et al., 2004). The expression of *NbRPL12* and *NbRPL19* was induced upon host or non-host pathogen infection (Nagaraj et al., 2016). Our present findings in which we have also observed the increased expression of *RPL19* under drought and limited water conditions after 3rd day of exposure are in accordance with these reports. The transcript level of *RPL19* was also increased in high expression lines of *RPL23A*. The RPL23A in association with RPL19 and other RPL proteins might be responsible for conferring tolerance under stress. Also, other RPL genes, such as *RPL33* had conferred tolerance to cold stress in tobacco (Rogalski et al., 2008). We have also noticed the continuous upregulation of *RPL33* during drought and limited water experiments.

The *RPL23A* transgenic plants in the T_2_ generation were categorized into low and high expression lines based on the transcript levels of the gene. A total of three high and seven low expression lines were identified, of which two high expression lines were selected to validate their resistance responses in WUE and tolerance to simulated drought and salt stresses. The Δ^13^C, which is a proxy for WUE, in selected transgenic plants was significantly lower than NS, indicating that *RPL23A* transgenic plants are water-use efficient. The selected lines also exhibited increased growth and productivity related parameters (such as tillering and seed yield) and also showed high chlorophyll fluorescence indicating an elevation in quantum efficiency compared with the NS under limited water conditions. The high expression of *RPL23A* is also accompanied by upregulation of other members of RPL and RPS genes, indicating that *RPL23A* works in close association with other RPs. The differential expression of other members of RP genes in two high expression lines of *RPL23A* suggested that the RPL and RPS genes are co-regulated in the cell. These results also highlight the possibility of cross-talk between RPL and RPS proteins.

The two high expression lines were progressed to T_3_ generation. An increased accumulation of osmolytes like proline helps plant to maintain the osmotic potential of the cell and confers tolerance to osmotic stresses (Kishor et al., 2014). Proline, not only acts as a free radical scavenger, but also protects the cell from damage that occurs during stress conditions (Hayat et al., 2012). It is apparent that the proline accumulation in *RPL23A* plants was higher than NC under PEG and NaCl stress treatments. The seedlings of high expression lines also displayed higher amounts of chlorophyll contents (Chl-a, -b, and total chlorophyll) than NS. The chlorophyll contents of NS decreased after treatment with stress-inducing agents like PEG and NaCl, but the transgenic seedlings continued to maintain elevated levels of these photosynthetic pigments.

Plants respond to the dehydration stress by closing their stomatal aperture to avoid transpirational water loss, which diminishes the photosynthesis (Hummel et al., 2010). The increase in chlorophyll fluorescence with high chlorophyll contents under stress conditions in *RPL23A* transgenics are most likely associated with increased photosynthetic activities under limited water availability and other stresses. The Arabidopsis RPL23aA knockout mutants exhibited retarded growth and development with perturbed phenotype (Degenhardt and Bonham-Smith, 2008). Hence, overexpression of *RPL23A* might have resulted in transgenic plants with increased biomass and yield.

In conclusion, our present and previous studies showed that RP genes, in addition to their universal roles of stabilizing the ribosomal complex and mediating polypeptide synthesis, also have extra-ribosomal functions, such as their involvement in response to the environmental stresses, such as dehydration.

## Author contributions

MM, PK, and MSM designed the experiments. MM performed all the experiments. AB helped in the analysis of qRT-PCR and physiological experiments. MSM organized the qRT-PCR studies and analysis with the second reference gene, tubulin as desired by the referees. MM and PK prepared the manuscript. MM, AB, MSM, and PK read and approved the manuscript.

### Conflict of interest statement

The authors declare that the research was conducted in the absence of any commercial or financial relationships that could be construed as a potential conflict of interest.

## Abbreviations

RP: Ribosomal Protein
RPL: Ribosomal Protein Large subunit
RPS: Ribosomal Protein Small subunit
WUE: Water-Use Efficiency.
