# Supplementary material for: Expression Profiling of Ribosomal Protein Gene Family in Dehydration Stress Responses and Characterization of Transgenic Rice Plants Overexpressing RPL23A for Water-Use Efficiency and Tolerance to Drought and Salt Stresses
Source: Front Chem. 2017 Nov 14;5:97. doi: 10.3389/fchem.2017.00097 (PMC5694489; doi:10.3389/fchem.2017.00097)
Supplement: Supplementary file 1 [file DataSheet1.DOCX]

**Supplementary Fig. 1. Growth of wild-type rice plants at the time of sampling under limited water and drought conditions**


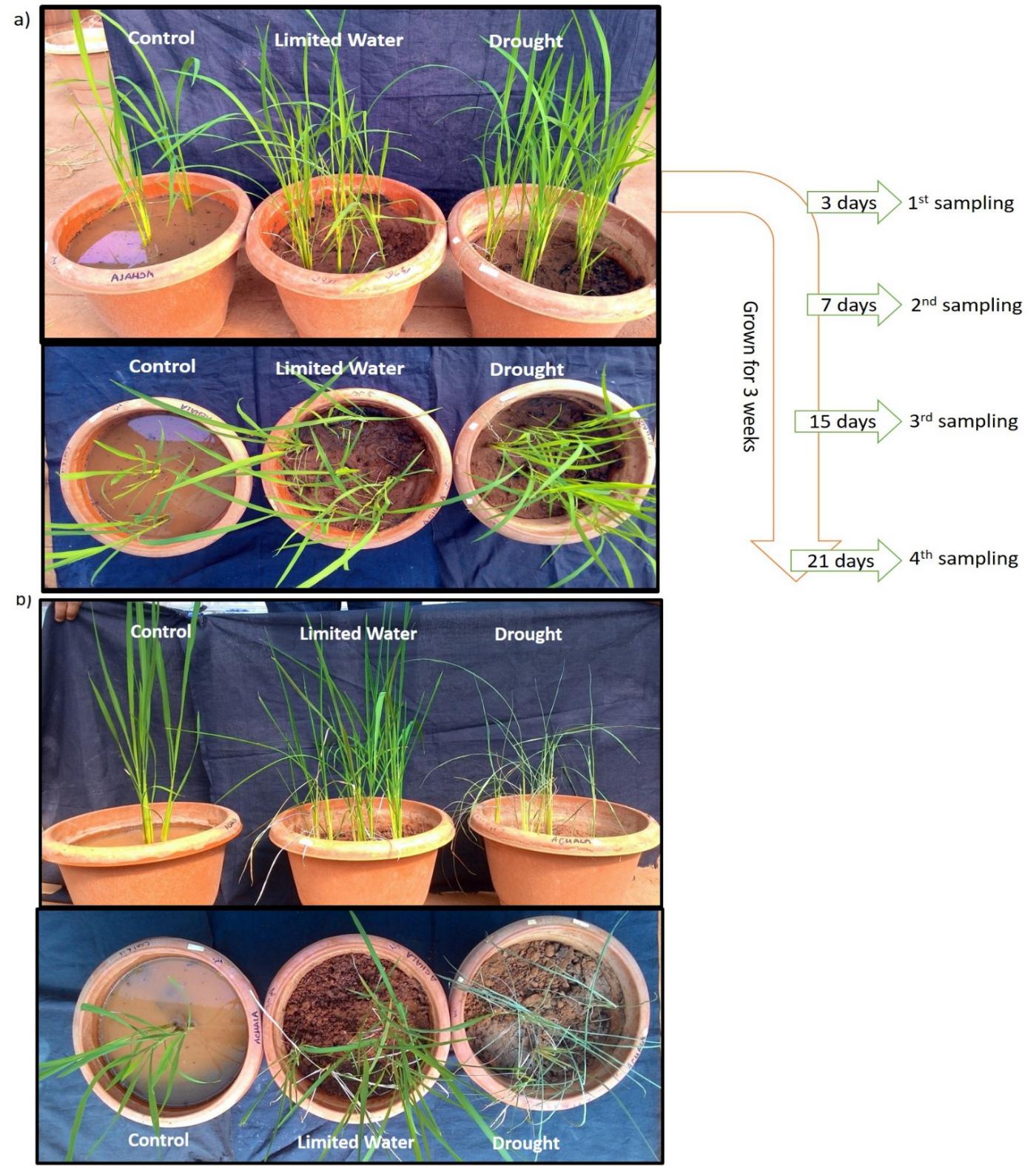


Fig. 1a. Growth of WT plants under limited water and drought conditions 3 d after treatment.

Fig. 1b. Water treatments were continued up to 21 d and samples were collected at intervals 3 d, 7 d, 15 d and 21 d after treatment.

**Supplementary Fig. 2. Graphical representation of expression pattern of RPL and RPS genes at different time intervals after exposure to limited water and drought conditions**


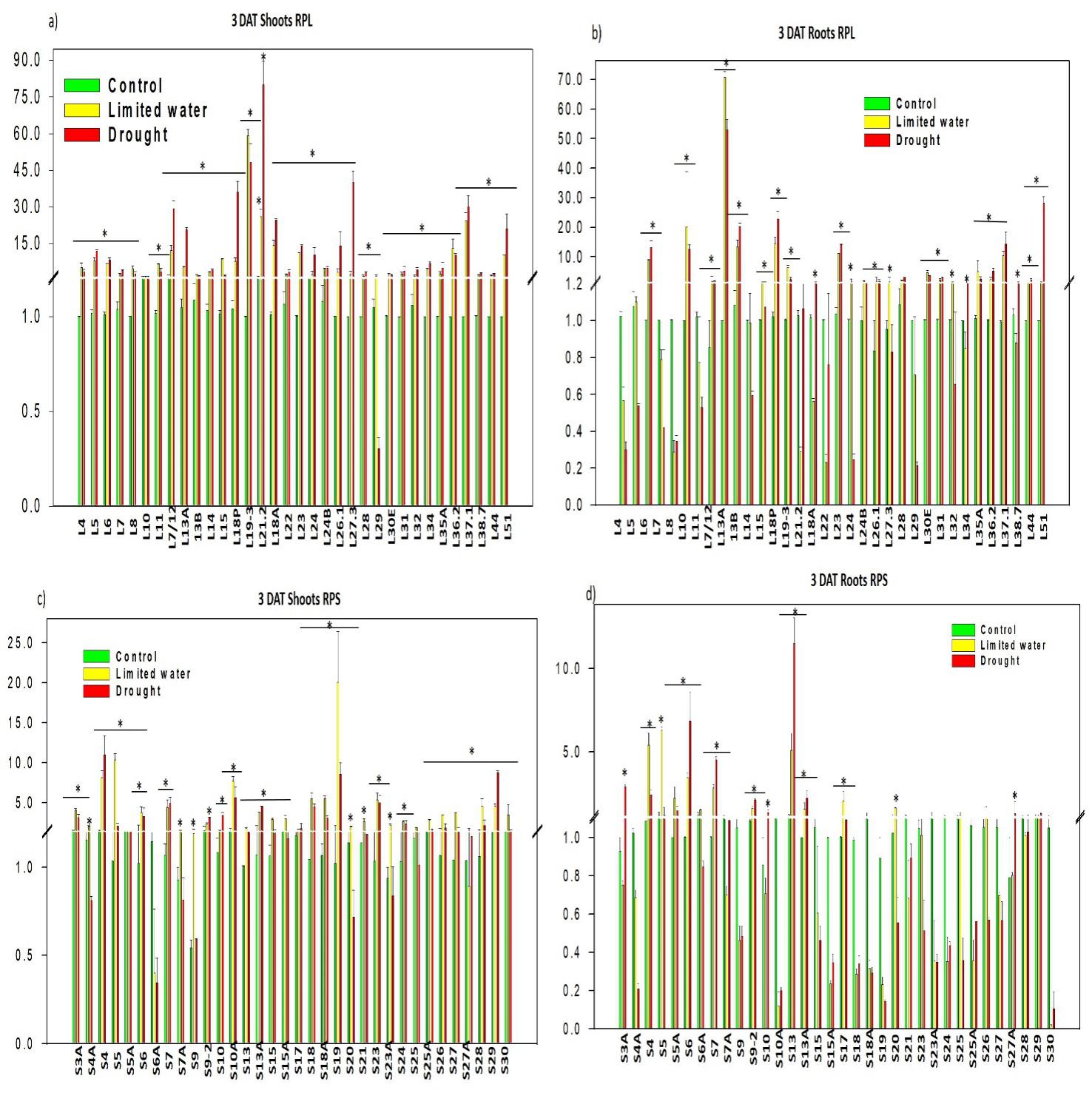


Expression pattern of RPL genes 3 d after treatment (DAT) in a) shoots and b) roots. Expression pattern of RPS genes 3 DAT in c) shoot and d) root tissues. Rice actin (*Act1*) and tubulin β-*tub* genes were used as an internal reference genes to normalize the expression (double normalization). The relative expression was considered statistically significant at *P* value <0.05 which is represented with asterisks in the graph based on one-way ANOVA.


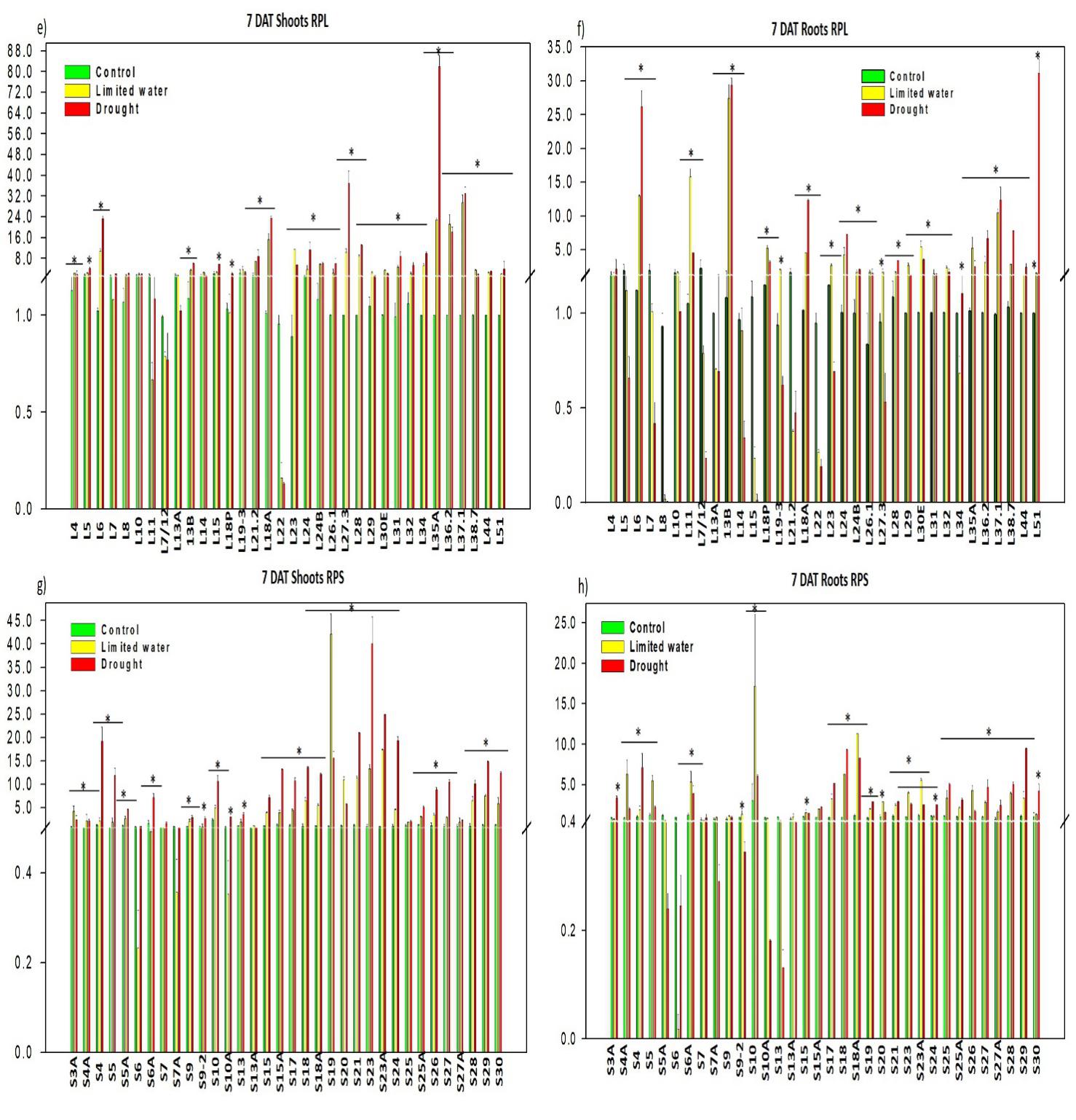


Expression pattern of RPL genes 7 d after treatment (DAT) in e) shoots and f) roots. Expression analysis of RPS genes 7 DAT in g) shoot and h) root tissues.


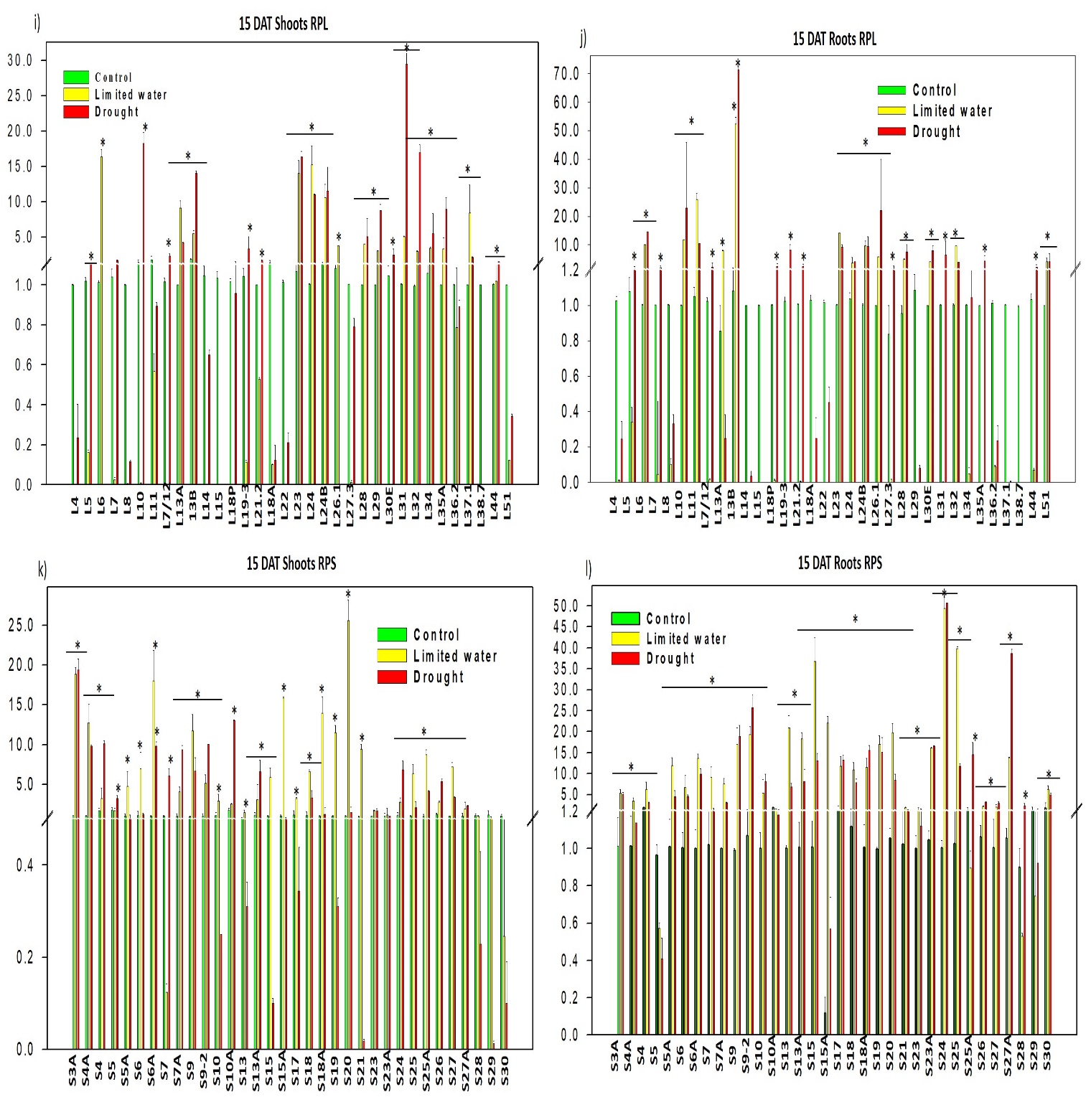


Expression pattern of RPL genes 15 d after treatment (DAT) in i) shoots and j) roots. Expression pattern of RPS genes 15 DAT in k) shoot and l) root tissues.


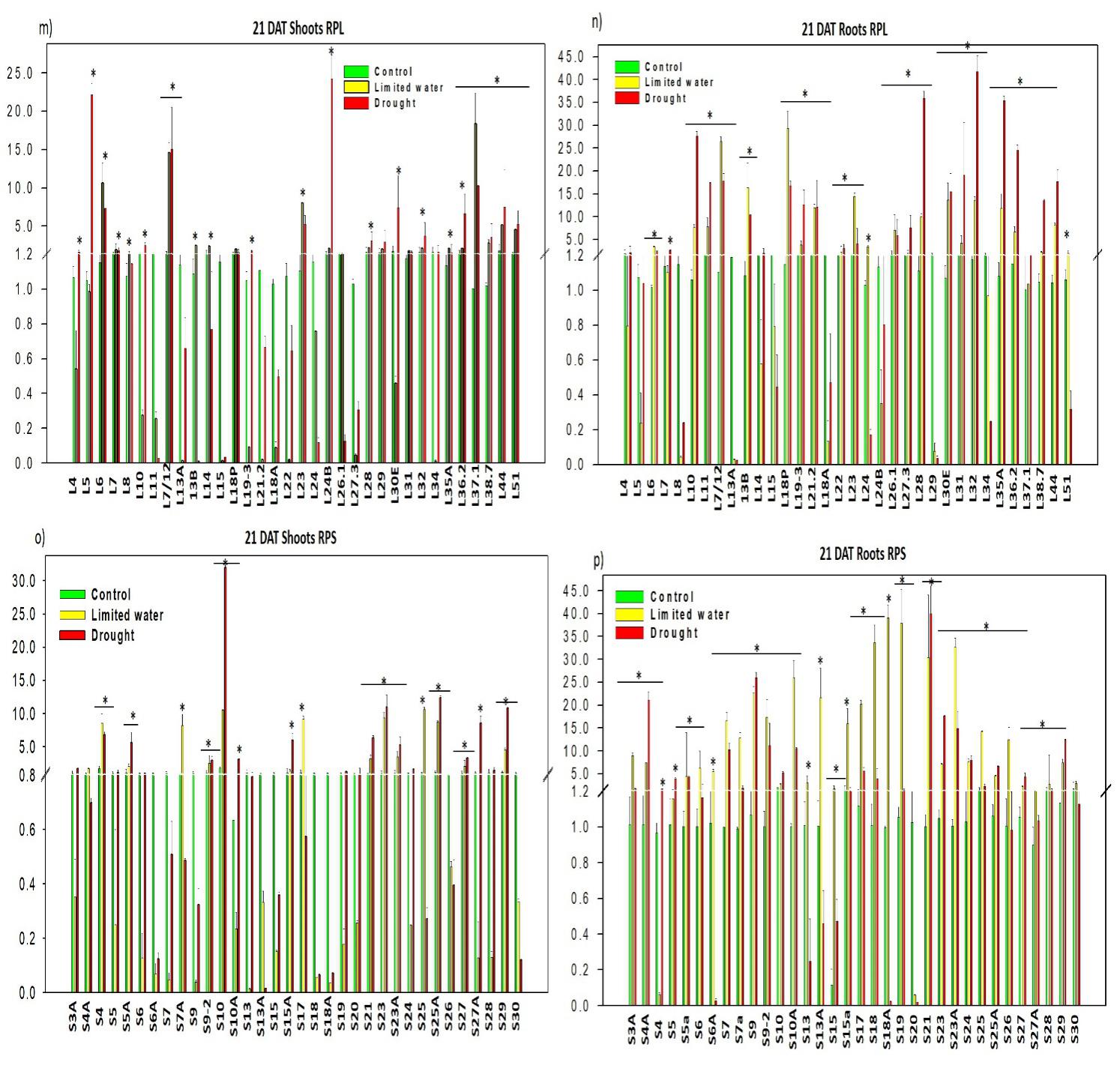


Expression pattern of RPL genes 21 d after treatment (DAT) in m) shoots and n) roots. Expression pattern of RPS genes 21 DAT in o) shoot and p) root tissues.

**The order of RPL genes presented in the graphs:** L4, L5, L6, L7, L8, L10, L11, L7/12, L13A, 13B, L14, L15, L18P, L19-3, L21.2, L18A, L22, L23, L24, L24B, L26.1, L27.3, L28, L29, L30E, L31, L32, L34, L35A, L36.2, L37.1, L38.7, L44, L51

**The order of RPS genes presented in the graphs:** S3A, S4A, S4, S5, S5a, S6, S6A, S7, S7a, S9, S9-2, S10, S10A, S13, S13A, S15, S15a, S17, S18, S18A, S19, S20, S21, S23, S23A, S24, S25, S25A, S26, S27, S27A, S28, S29, S30

**Supplementary Fig. 3**

**Overlap in the Up and down-regulation of rice RPL genes in response to limited water and drought conditions**

**
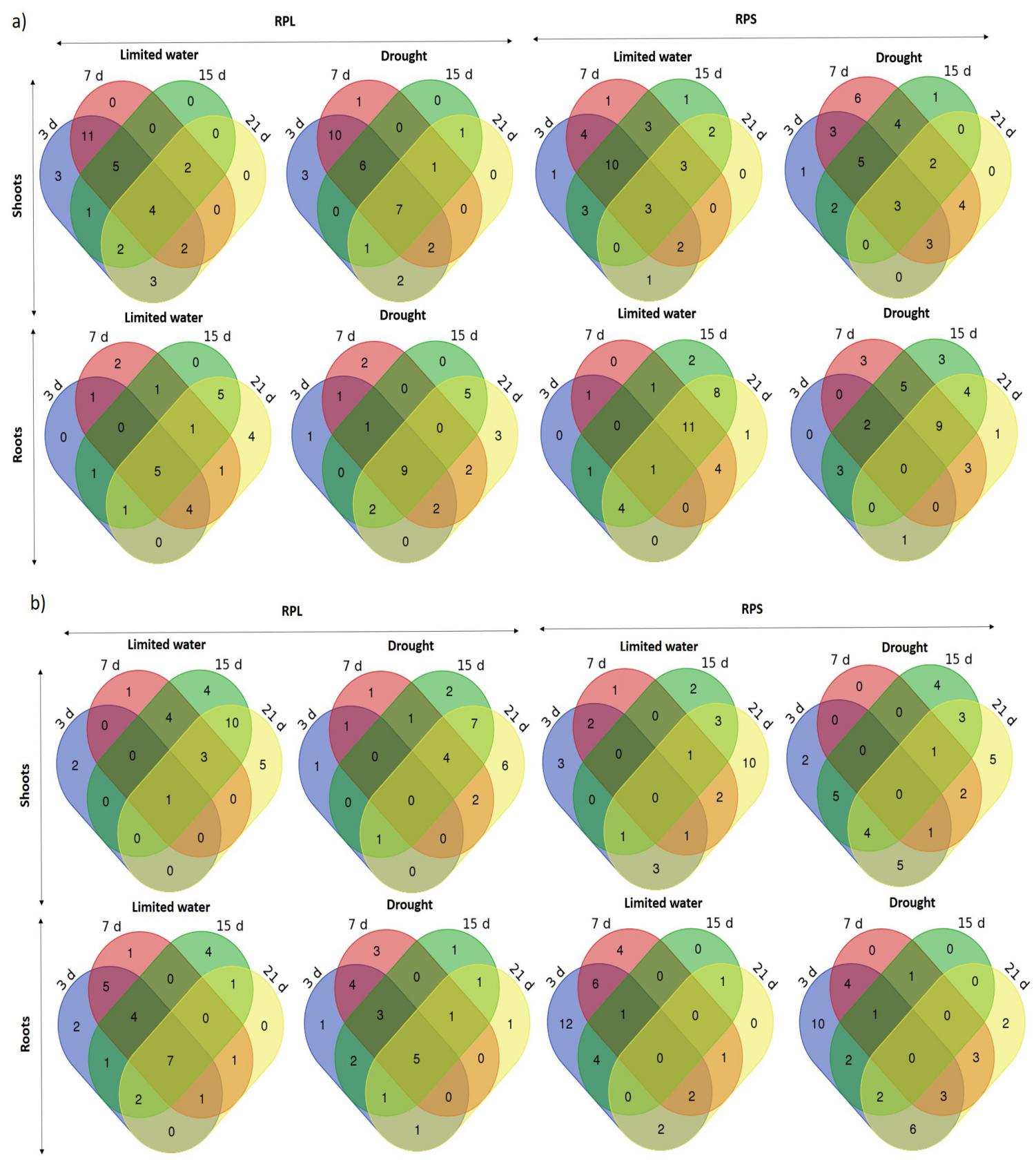
**

**Supplementary Fig. 3.** The RPL genes that exhibited >2-fold transcript level on the log_2_ scale were considered as up-regulated. Venn diagrams were used to represent the overlap in (a) up and (b) down-regulation in shoot and root tissues separately. The Venn diagrams that correspond to RPL and RPS genes were represented on the top.

**Supplementary Fig. 4**


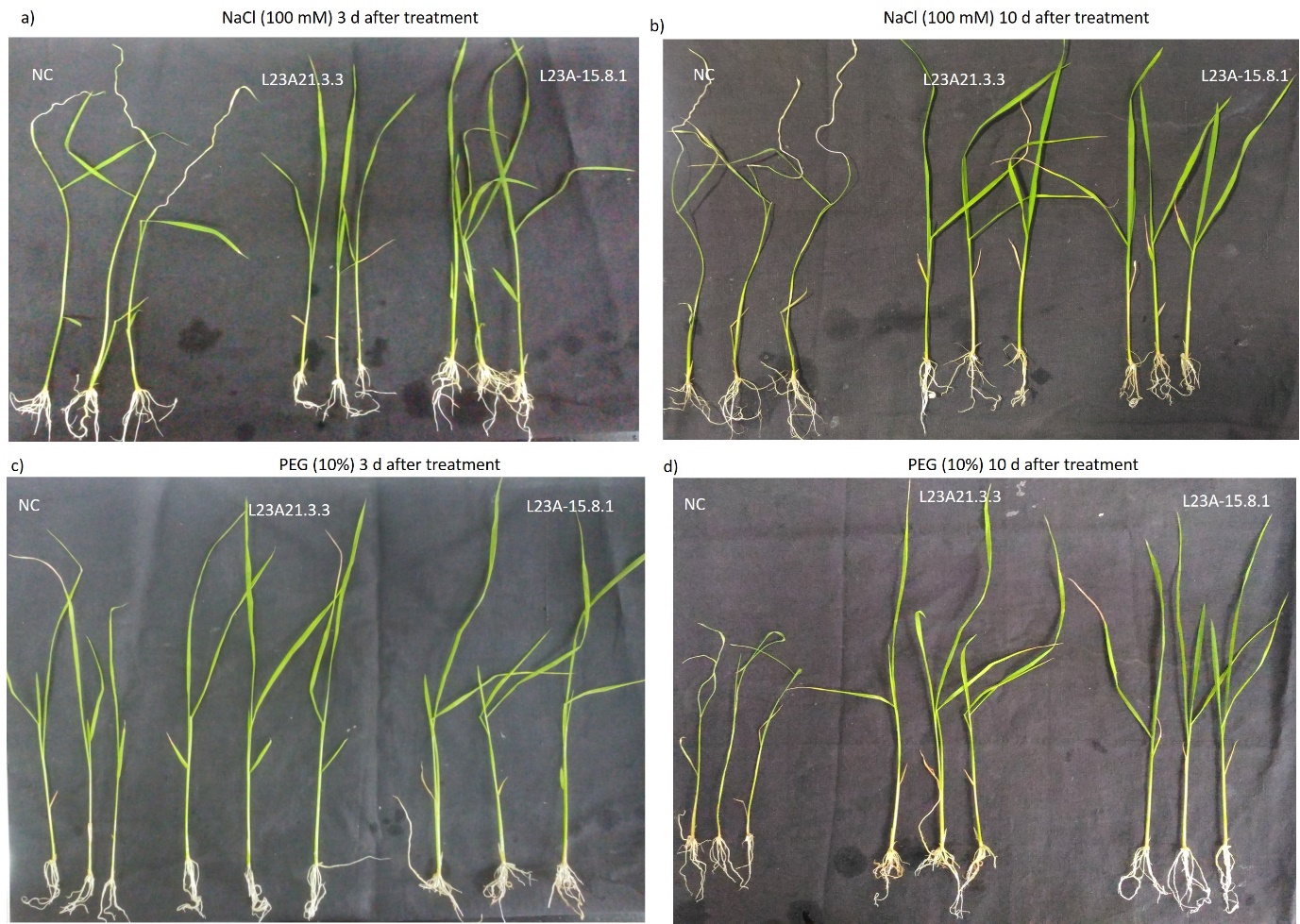


**Supplementary Fig. 4.** The two selected lines of T_3_ generation rice transgenic plants showed improved tolerance to mild levels of salt stress (NaCl) (a) 3 d and (b) 10 d after treatment. Similarly, tolerance was also observed in response to simulated drought (PEG) (c) 3 d and (d) 10 d after treatment in comparison with Null Segregant (NC or NS).

**Supplementary Fig. 5**

**
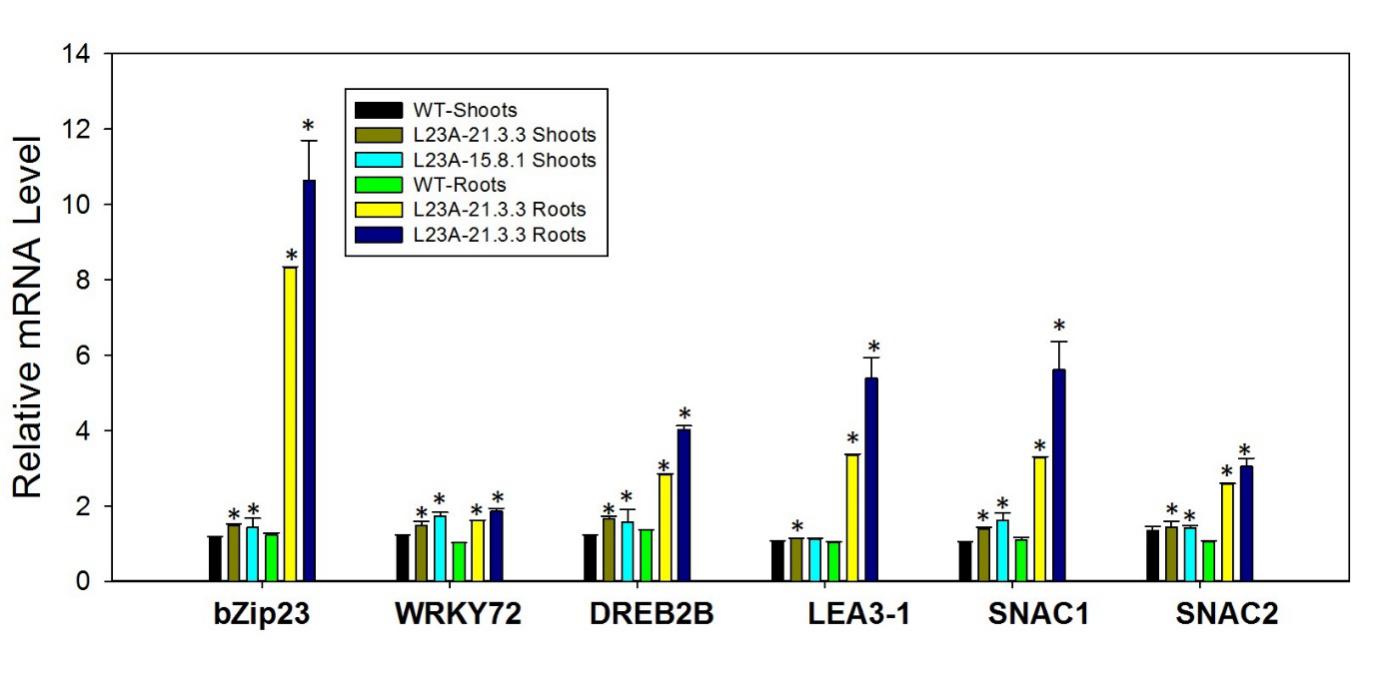
**

**Supplementary Fig. 5. Expression profiling of stress-specific genes in transgenic rice seedlings overexpressing *RPL23A***

All the genes became upregulated in both the lines, but significant upregulation was observed in roots than in shoots. Because the stress signals such as drought and salt are perceived through roots, high expression of stress-specific genes in roots is particularly important as they might inhibit the transmission of stress signal to aerial parts of the plant.

**Supplementary Table 1. Overlap in the upregulation and downregulation of RP genes at different time intervals after treatment with limited water and drought conditions in shoot and root tissues separately**

1. **Upregulated RPL genes in shoots in response to Limited water treatment**

| **Treatment time interval** | **Total no. of genes** | **Gene type** |
| --- | --- | --- |
| 15 d 21 d 3 d 7 d | 4 | L23 L6 L37.1 L32 |
| 15 d 3 d 7 d | 5 | L31 L35A L19-3 L34 13B |
| 21 d 3 d 7 d | 2 | L44 L38.7 |
| 15 d 21 d 3 d | 2 | L30E L12 |
| 15 d 21 d 7 d | 2 | L29 L28 |
| 3 d 7 d | 11 | L27.3 L24 L18A L5 L24B L4 L21.2 L15 L36.2 L26.1 L51 |
| 15 d 3 d | 1 | L13A |
| 21 d 3 d | 3 | L18P L14 L7 |
| 3 d | 3 | L11 L22 L8 |

1. **Upregulated RPL genes in Shoots under Drought treatment**

| **Treatment time interval** | **Total no. of genes** | | **Gene type** |
| --- | --- | --- | --- |
| 15 d 21 d 3 d 7 d | | 7 | L5 L28 L24B L23 L6 L37.1 L32 |
| 15 d 3 d 7 d | | 6 | L31 L18P L35A L19-3 L34 13B |
| 21 d 3 d 7 d | | 2 | L44 L30E |
| 15 d 21 d 3 d | | 1 | L13A |
| 15 d 21 d 7 d | | 1 | L29 |
| 3 d 7 d | | 10 | L27.3 L14 L24 L18A L21.2 L8 L36.2 L26.1 L51 L7 |
| 21 d 3 d | | 2 | L38.7 L12 |
| 15 d 21 d | | 1 | L10 |
| 3 d | | 3 | L11 L22 L4 |
| 7 d | | 1 | L15 |

1. **Upregulated RPL genes in roots under Limited water**

| **Treatment time interval** | **Total no. of genes** | | | **Gene type** |
| --- | --- | --- | --- | --- |
| 15 d 21 d 3 d 7 d | | 5 | L18P L30E L23 13B L6 | |
| 21 d 3 d 7 d | | 4 | L31 L19-3 L36.2 L35A | |
| 15 d 21 d 3 d | | 1 | L10 | |
| 15 d 21 d 7 d | | 1 | L24 | |
| 3 d 7 d | | 1 | L37.1 | |
| 15 d 3 d | | 1 | L13A | |
| 15 d 7 d | | 1 | L24B | |
| 21 d 7 d | | 1 | L38.1 | |
| 15 d 21 d | | 5 | L28 L11 L26.1 L51 L32 | |
| 7 d | | 2 | L29 L18A | |
| 21 d | | 4 | L44 L27.3 L21.2 L22 | |

1. **Upregulated RPL genes in roots under Drought**

| **Treatment time interval** | **Total no. of genes** | | | **Gene type** |
| --- | --- | --- | --- | --- |
| 15 d 21 d 3 d 7 d | | 9 | L28 L31 L18P L30E L23 13B L6 L24 L35A | |
| 15 d 3 d 7 d | | 1 | L51 | |
| 21 d 3 d 7 d | | 2 | L44 L36.2 | |
| 15 d 21 d 3 d | | 2 | L19-3 L10 | |
| 3 d 7 d | | 1 | L37.1 | |
| 21 d 7 d | | 2 | L38.1 L4 | |
| 15 d 21 d | | 5 | L11 L21.2 L26.1 L7 L32 | |
| 3 d | | 1 | L13A | |
| 7 d | | 2 | L24B L18A | |
| 21 d | | 3 | L27.3 L14 L22 | |

1. **Upregulated RPS genes in shoots under Limited water**

| **Treatment time interval** | **Total no. of genes** | | **Gene type** |
| --- | --- | --- | --- |
| 15 d 21 d 3 d 7 d | | 3 | RPS23A S4 RPS27 |
| 15 d 3 d 7 d | | 10 | S15a S19 S18A RPS25A RPS26 S18 S4A S3A S15 RPS24 |
| 21 d 3 d 7 d | | 2 | S29 RPS23 |
| 15 d 21 d 7 d | | 3 | S10 S5a S17 |
| 3 d 7 d | | 4 | S30 S28 RPS21 S5 |
| 15 d 3 d | | 3 | S10A S6 S13A |
| 21 d 3 d | | 1 | S9-2 |
| 15 d 7 d | | 3 | S20 RPS27A S9 |
| 15 d 21 d | | 2 | S25 S7a |
| 3 d | | 1 | S7 |
| 7 d | | 1 | S13 |
| 15 d | | 1 | S6A |

1. **Upregulated RPS genes in shoots under Drought**

| **Treatment time interval** | **Total no. of genes** | | **Gene type** |
| --- | --- | --- | --- |
| 15 d 21 d 3 d 7 d | | 3 | S10A S9-2 S4 |
| 15 d 3 d 7 d | | 5 | RPS26 S5 S18 S3A RPS24 |
| 21 d 3 d 7 d | | 3 | S29 S10 RPS23 |
| 15 d 21 d 7 d | | 2 | RPS23A RPS27A |
| 3 d 7 d | | 3 | S19 S18A S28 |
| 15 d 3 d | | 2 | S13A S7 |
| 15 d 7 d | | 4 | S6A S9 S25 S4A |
| 21 d 7 d | | 4 | S15a RPS25A S5a RPS27 |
| 3 d | | 1 | S6 |
| 7 d | | 6 | S30 S13 S20 RPS21 S17 S15 |
| 15 d | | 1 | S7a |

1. **Upregulated RPS genes in roots under Limited water**

| **Treatment time interval** | **Total no. of genes** | | **Gene type** |
| --- | --- | --- | --- |
| 15 d 21 d 3 d 7 d | | 1 | S17 |
| 15 d 21 d 3 d | | 4 | S13 S5a S6 S7 |
| 15 d 21 d 7 d | | 11 | S15a RPS23A S19 S18A S6A S10 S25 RPS26 S18 S4A RPS27 |
| 3 d 7 d | | 1 | S5 |
| 15 d 3 d | | 1 | S4 |
| 15 d 7 d | | 1 | S20 |
| 21 d 7 d | | 4 | S29 S28 RPS25A RPS21 |
| 15 d 21 d | | 8 | S30 S9-2 S9 S7a RPS23 S3A RPS24 S13A |
| 15 d | | 2 | RPS27A S15 |
| 21 d | | 1 | S10A |

1. **Upregulated RPS genes in roots under Drought**

| **Treatment time interval** | **Total no. of genes** | | **Gene type** |
| --- | --- | --- | --- |
| 15 d 3 d 7 d | | 2 | S4 S3A |
| 15 d 21 d 7 d | | 9 | RPS23A S18A S10 S25 RPS25A S18 S17 RPS24 RPS27 |
| 15 d 3 d | | 3 | S13 S6 S13A |
| 21 d 3 d | | 1 | S7 |
| 15 d 7 d | | 5 | S30 S19 S6A S28 RPS26 |
| 21 d 7 d | | 3 | S29 RPS21 S4A |
| 15 d 21 d | | 4 | S9-2 S9 S7a RPS23 |
| 7 d | | 3 | S15a RPS27A S5 |
| 15 d | | 3 | S20 S5a S15 |
| 21 d | | 1 | S10A |

1. **Downregulated RPL genes in shoots under Limited water**

| **Treatment time interval** | **Total no. of genes** | **Gene type** |
| --- | --- | --- |
| 15 d 21 d 3 d 7 d | 1 | L10 |
| 15 d 21 d 7 d | 3 | L11 L8 L22 |
| 15 d 7 d | 4 | L18P L30E L12 L7 |
| 15 d 21 d | 10 | L5 L27.3 L24B L4 L21.2 L36.2 L15 L26.1 L24 L18A |
| 3 d | 2 | L28 L29 |
| 7 d | 1 | L13A |
| 15 d | 4 | L44 L14 L51 L38.7 |
| 21 d | 5 | L31 L19-3 L34 13B L35A |

1. **Downregulated RPL genes in shoots under Drought**

| **Treatment time interval** | **Total no. of genes** | **Gene type** |
| --- | --- | --- |
| 15 d 21 d 3 d | 1 | L15 |
| 15 d 21 d 7 d | 4 | L11 L4 L14 L22 |
| 3 d 7 d | 1 | L10 |
| 15 d 7 d | 1 | L38.7 |
| 21 d 7 d | 2 | L8 L13A |
| 15 d 21 d | 7 | L27.3 L21.2 L36.2 L26.1 L24 L7 L18A |
| 3 d | 1 | L29 |
| 7 d | 1 | L12 |
| 15 d | 2 | L44 L51 |
| 21 d | 6 | L31 L18P L19-3 L34 13B L35A |

1. **Downregulated RPL genes in roots under Limited water**

| **Treatment time interval** | **Total no. of genes** | **Gene type** |
| --- | --- | --- |
| 15 d 21 d 3 d 7 d | 7 | L5 L4 L34 L14 L8 L15 L7 |
| 15 d 3 d 7 d | 4 | L44 L27.3 L21.2 L22 |
| 21 d 3 d 7 d | 1 | L24B |
| 15 d 21 d 3 d | 2 | L29 L18A |
| 3 d 7 d | 5 | L28 L26.1 L51 L12 L32 |
| 15 d 3 d | 1 | L38.7 |
| 21 d 7 d | 1 | L13A |
| 15 d 21 d | 1 | L37.1 |
| 3 d | 2 | L11 L24 |
| 7 d | 1 | L10 |
| 15 d | 4 | L31 L19-3 L36.2 L35A |

1. **Downregulated RPL genes in roots under Drought**

| **Treatment time interval** | **Total no. of genes** | **Gene type** |
| --- | --- | --- |
| 15 d 21 d 3 d 7 d | 5 | L5 L29 L34 L8 L15 |
| 15 d 3 d 7 d | 3 | L27.3 L14 L22 |
| 15 d 21 d 3 d | 1 | L18A |
| 15 d 21 d 7 d | 1 | L13A |
| 3 d 7 d | 4 | L21.2 L26.1 L7 L32 |
| 15 d 3 d | 2 | L4 L38.7 |
| 21 d 3 d | 1 | L24B |
| 15 d 21 d | 1 | L37.1 |
| 3 d | 1 | L11 |
| 7 d | 3 | L19-3 L10 L12 |
| 15 d | 1 | L36.2 |
| 21 d | 1 | L51 |

1. **Downregulated RPS genes in shoots under Limited water**

| **Treatment time interval** | **Total no. of genes** | **Gene type** |
| --- | --- | --- |
| 21 d 3 d 7 d | 1 | S6A |
| 15 d 21 d 3 d | 1 | S13 |
| 15 d 21 d 7 d | 1 | S7 |
| 3 d 7 d | 2 | S25 S7a |
| 21 d 3 d | 3 | S9 S20 RPS27A |
| 21 d 7 d | 2 | S6 S13A |
| 15 d 21 d | 3 | S30 S28 S5 |
| 3 d | 3 | S10 S5a S17 |
| 7 d | 1 | S9-2 |
| 15 d | 2 | S29 RPS23 |
| 21 d | 10 | S15a RPS26 S18 S10A S4A S19 S3A S15 RPS24 S18A |

1. **Downregulated RPS genes in shoots under Drought**

| **Treatment time interval** | **Total no. of genes** | **Gene type** |
| --- | --- | --- |
| 21 d 3 d 7 d | 1 | S7a |
| 15 d 21 d 3 d | 4 | S30 S13 S20 S17 |
| 15 d 21 d 7 d | 1 | S6 |
| 15 d 3 d | 5 | S15a RPS25A RPS21 S5a RPS27 |
| 21 d 3 d | 5 | S6A S9 S25 S4A S15 |
| 21 d 7 d | 2 | S7 S13A |
| 15 d 21 d | 3 | S19 S18A S28 |
| 3 d | 2 | RPS23A RPS27A |
| 15 d | 4 | S29 S9-2 S10 RPS23 |
| 21 d | 5 | RPS26 S5 S18 S3A RPS24 |

1. **Downregulated RPS genes in roots under Limited water**

| **Treatment time interval** | **Total no. of genes** | **Gene type** |
| --- | --- | --- |
| 15 d 3 d 7 d | 1 | S10A |
| 21 d 3 d 7 d | 2 | RPS27A S15 |
| 3 d 7 d | 6 | S30 S9-2 S9 S7a RPS24 S13A |
| 15 d 3 d | 4 | S29 S28 RPS25A RPS21 |
| 21 d 3 d | 2 | S20 S4A |
| 21 d 7 d | 1 | S4 |
| 15 d 21 d | 1 | S5 |
| 3 d | 12 | S15a RPS23A S19 S18A S6A S10 S25 RPS26 RPS23 S18 S3A RPS27 |
| 7 d | 4 | S13 S5a S6 S7 |

1. **Downregulated RPS genes in roots under Drought**

| **Treatment time interval** | **Total no. of genes** | **Gene type** |
| --- | --- | --- |
| 15 d 3 d 7 d | 1 | S10A |
| 21 d 3 d 7 d | 3 | S20 S5a S15 |
| 15 d 21 d 3 d | 2 | S15a S5 |
| 3 d 7 d | 4 | S9-2 S9 S7a RPS23 |
| 15 d 3 d | 2 | S29 RPS21 |
| 21 d 3 d | 6 | S30 S19 RPS27A S6A S28 RPS26 |
| 15 d 7 d | 1 | S7 |
| 21 d 7 d | 3 | S13 S6 S13A |
| 3 d | 10 | RPS23A S18A S10 S25 RPS25A S18 S17 S4A RPS24 RPS27 |
| 21 d | 2 | S4 S3A |

**Supplementary Table 2. Quantum efficiency and panicle characters in transgenic**

**rice plants overexpressing *RPL23A***

| Plant | Number of tillers | Number of panicles | Panicle length (cm) | Seed yield (g) | Quantum efficiency (*Fv/Fm)* |
| --- | --- | --- | --- | --- | --- |
| NC | 5±0.613 | 3±0.462 | 8±0.312 | 8±0.571 | 0.650±0.162 |
| L23A-15.8.1 | 13±0.817 | 9±0.715 | 12±0.412 | 21±0.527 | 0.801±0.173 |
| L23A-12.6.22 | 12±0.628 | 8±0.590 | 10±0.452 | 20±0.638 | 0.803±0.126 |
